# Sleep deprivation impairs cognitive performance, alters task-associated cerebral blood flow and decreases cortical neurovascular coupling-related hemodynamic responses

**DOI:** 10.1038/s41598-021-00188-8

**Published:** 2021-10-25

**Authors:** Tamas Csipo, Agnes Lipecz, Cameron Owens, Peter Mukli, Jonathan W. Perry, Stefano Tarantini, Priya Balasubramanian, Ádám Nyúl-Tóth, Valeriya Yabluchanska, Farzaneh A. Sorond, J. Mikhail Kellawan, György Purebl, William E. Sonntag, Anna Csiszar, Zoltan Ungvari, Andriy Yabluchanskiy

**Affiliations:** 1 Vascular Cognitive Impairment and Neurodegeneration Program, Oklahoma Center for Geroscience and Healthy Brain Aging, Department of Biochemistry and Molecular Biology, University of Oklahoma Health Sciences Center, 975 NE 10th Street, BRC 1301, Oklahoma City, OK 73104 USA; 2 Division of Clinical Physiology, Department of Cardiology, Faculty of Medicine, University of Debrecen, Debrecen, Hungary; 3 International Training Program in Geroscience, Doctoral School of Basic and Translational Medicine/Department of Public Health, Semmelweis University, Budapest, Hungary; 4 Department of Ophthalmology, Josa Andras Hospital, Nyíregyháza, Hungary; 5 Department of Health and Exercise Science, University of Oklahoma, Norman, OK USA; 6 International Training Program in Geroscience, Department of Physiology, Faculty of Medicine, Semmelweis University, Budapest, Hungary; 7 Division of Stroke and Neurocritical Care, Department of Neurology, Northwestern University Feinberg School of Medicine, Chicago, IL USA; 8 Institute of Behavioral Sciences, Semmelweis University, Budapest, Hungary; 9 International Training Program in Geroscience, Theoretical Medicine Doctoral School/Departments of Cell Biology and Molecular Medicine and Medical Physics and Informatics, University of Szeged, Szeged, Hungary; 10 Department of Health Promotion Sciences, College of Public Health, University of Oklahoma Health Sciences Center, Oklahoma City, OK USA

**Keywords:** Translational research, Neurological disorders, Cognitive neuroscience

## Abstract

Sleep deprivation (SD) is a common condition and an important health concern. In addition to metabolic and cardiovascular risks, SD associates with decreases in cognitive performance. Neurovascular coupling (NVC, "functional hyperemia") is a critical homeostatic mechanism, which maintains adequate blood supply to the brain during periods of intensive neuronal activity. To determine whether SD alters NVC responses and cognitive performance, cognitive and hemodynamic NVC assessments were conducted prior to and 24 h post-SD in healthy young male individuals (*n* = 10, 27 ± 3 years old). Cognition was evaluated with a battery of tests from the Cambridge Neuropsychological Test Automated Battery (CANTAB). Hemodynamic components of NVC were measured by transcranial Doppler sonography (TCD) during cognitive stimulation, dynamic retinal vessel analysis (DVA) during flicker light stimulation, and functional near infrared spectroscopy (fNIRS) during finger tapping motor task. Cognitive assessments revealed impairments in reaction time and sustained attention after 24 h of SD. Functional NIRS analysis revealed that SD significantly altered hemodynamic responses in the prefrontal cortex and somatosensory cortex during a motor task. NVC-related vascular responses measured by DVA and TCD did not change significantly. Interestingly, TCD detected decreased task-associated cerebral blood flow (CBF) in the right middle cerebral artery in sleep deprived participants. Our results demonstrate that 24 h of SD lead to impairments in cognitive performance together with altered CBF and hemodynamic components of cortical NVC responses.

## Introduction

Sleep disorders recently emerged as one of the top health concerns of the twenty-first century^1^, as chronic insomnia itself affects approximately 10% of the population^2^. According to recent reports from the National Sleep Foundation, appropriate sleep duration should constitute between 7 to 9 h for young and middle aged adults of 18 to 65 years of age^3^. Every sleep duration that is less than 7 h is considered a short sleep duration. Centers for Chronic Disease Control and Prevention report that prevalence of short sleep duration among young and middle-aged adults ranges from 32 to 39%^4^. In addition to elevated risks of chronic somatic diseases^5^, short sleep duration is strongly associated with decreases in cognitive performance^6^, which may have a significant implication on work productivity and incidence of accidental injuries in a working class of adults. These effects do not necessarily occur only as a result of chronic sleep deprivation, as a recent meta-analytic review concluded that impairments in several cognitive domains can be observed even after short-term sleep restriction^7^. Loss of sleep may affect several domains of cognitive functioning, however, reaction time and working memory are reported to be the most sensitive to short-term sleep deprivation^8–10^. Working memory is the cognitive domain that is responsible for temporarily holding information available for manipulating, processing, and is also involved in the transition of information to long-term memory^9,11^. Appropriate amount and quality of sleep is, therefore, essential for healthy neurocognitive function and memory consolidation, and lack of sleep is recognized to be associated with the increased risk of various neurocognitive disorders^12^. Further, a recent study suggested that 24 h of sleep deprivation may serve as an Alzheimer’s disease (AD) challenge model, as sleep deprivation-associated impairments in cognitive function were moderated by commonly prescribed AD drugs^10^.

Sleep disturbance and cognitive impairment also have a complex bidirectional relationship, and better understanding of the underlying pathophysiological mechanisms may provide a potential for the prevention and treatment of dementia and other cognitive disorders^13^.

Normal brain function is critically dependent on continuous and adequate blood supply. During periods of intensive neuronal activity, the additional neuronal demand of nutrients is matched by increased blood flow via neurovascular coupling (NVC). Recent preclinical studies demonstrate that impairment of NVC-associated hemodynamic responses is associated with impairments in cognitive performance and alterations in motor function^14,15^.

NVC-associated hyperemia, the “functional hyperemia” of the brain provides the basis for many noninvasive neuroimaging methods in humans, including functional magnetic resonance imaging (fMRI) and functional near infrared spectroscopy (fNIRS). However, users of these methods often consider NVC-associated signals only as a marker of neuronal activation and often assume that magnitude of signals originated from hemodynamic changes are constant at similar levels of neuronal activation. Hemodynamic consequences of NVC can also be assessed in the cerebral vasculature, more distal to the brain cortex. For example, transcranial Doppler sonography (TCD) is a method commonly used to assess NVC-associated hemodynamic changes in supplying arteries of different major cortical regions (e. g. in the posterior or middle cerebral artery)^16,17^, and its ability to sensitively detect changes in NVC in response to interventions has been recently demonstrated^18^. Both TCD and fNIRS are also capable of measuring the hemodynamic components of NVC at different levels of the vasculature. TCD detects changes in arterial blood flow in a major artery that supplies large areas of the brain, including those, where neuronal activation and consequential hyperemia is expected in response to cognitive stimulation. fNIRS detects changes in hemoglobin concentration in smaller areas of the brain cortex, which can be used to identify differential activation of specific areas, and similarly to TCD, it also provides an indirect measure of microcirculatory blood flow. Indirect measurement of cerebral hemodynamics via TCD or fNIRS can be informative of changes in diameter of smaller cerebrovascular vessels, however, they may be influenced by other, confounding factors as well (e. g. stenosis of a major artery may also cause increased flow velocity during TCD examination). Another recently developed technique, dynamic vessel analysis (DVA), allows direct measurement of microvascular diameter in the central nervous system (CNS). The DVA methodology has demonstrated great capabilities of measuring NVC-associated changes in microvascular diameter in response to flicker light stimulation^19,20^. This response, compared to responses recorded with TCD or fNIRS, is a form of NVC with fewer confounders, as the stimulus (flicker light) can be easily standardized, and the evoked activation of a neuronal population is more localized.

To date, there is very limited research done to understand the effect of sleep deprivation (SD) on NVC-associated hyperemia, and the present study was designed to test whether cognitive deficits associate with impaired CNS hemodynamic responses after SD. To achieve this goal, we evaluated the effects of 24-h SD on several domains of cognitive function and assessed NVC-related hemodynamic responses. We also aimed to determine whether the hemodynamic impairment is generally present and detectable by several methods while different modalities of stimulation are applied. Therefore, cerebrovascular hemodynamic changes were measured in young healthy adults before and after 24 h of sleep deprivation using fNIRS during a simple motor task, TCD during working memory test, and DVA in response to flicker light stimulation.

## Materials and methods

### Study participants and study design

A total of ten healthy young adults (males, 1 left-handed) were recruited for participation in this study (age: 27.6 ± 3.7 years, BMI: 22.6 ± 8.2 kg/m^2^). All enrolled individuals had a college degree. Informed consent was obtained prior to participation in the study, and the protocol was approved by the Institutional Review Board of the University of Oklahoma Health Sciences Center. All assessments were performed in accordance with relevant guidelines and regulations.

None of the participants reported neurological, psychiatric diseases, or any other significant medical condition (cardiovascular disease, cancer, diabetes mellitus, infection within 2 weeks of the examination date) or behavioral risk factors such as smoking, drug abuse, and excessive alcohol consumption. Study participants were screened for previous symptoms of glaucoma attack, history of photosensitive epilepsy, prior eye surgeries and retinal laser coagulation, severe cataracts, progressive diabetic retinopathy, allergies to tropicamide and tetracaine.

All participants kept a diary log for a duration of 7 days prior to the day of sleep deprivation, recording approximate time of falling asleep, time it took to fall asleep, number of times participants woke up at night, time of final awakening in the morning, events during these days that could affect sleep (including illness, emotional stress, disturbances, etc.), amount of consumed caffeinated and alcoholic beverages, and average sleepiness on a scale of 1–10. All participating subjects were requested to maintain a sleep duration of no less than 7 uninterrupted hours daily over the course of 7 days.

All baseline (pre-SD) measures were collected 3–5 days prior to the experimental day of sleep deprivation. On the day of sleep deprivation, subjects arrived at the testing facility where they remained overnight until the following morning when the final assessments were performed. Study personnel was present in the testing facility to ensure adherence to the protocol. None of the subjects were excluded.

**Figure 1:**
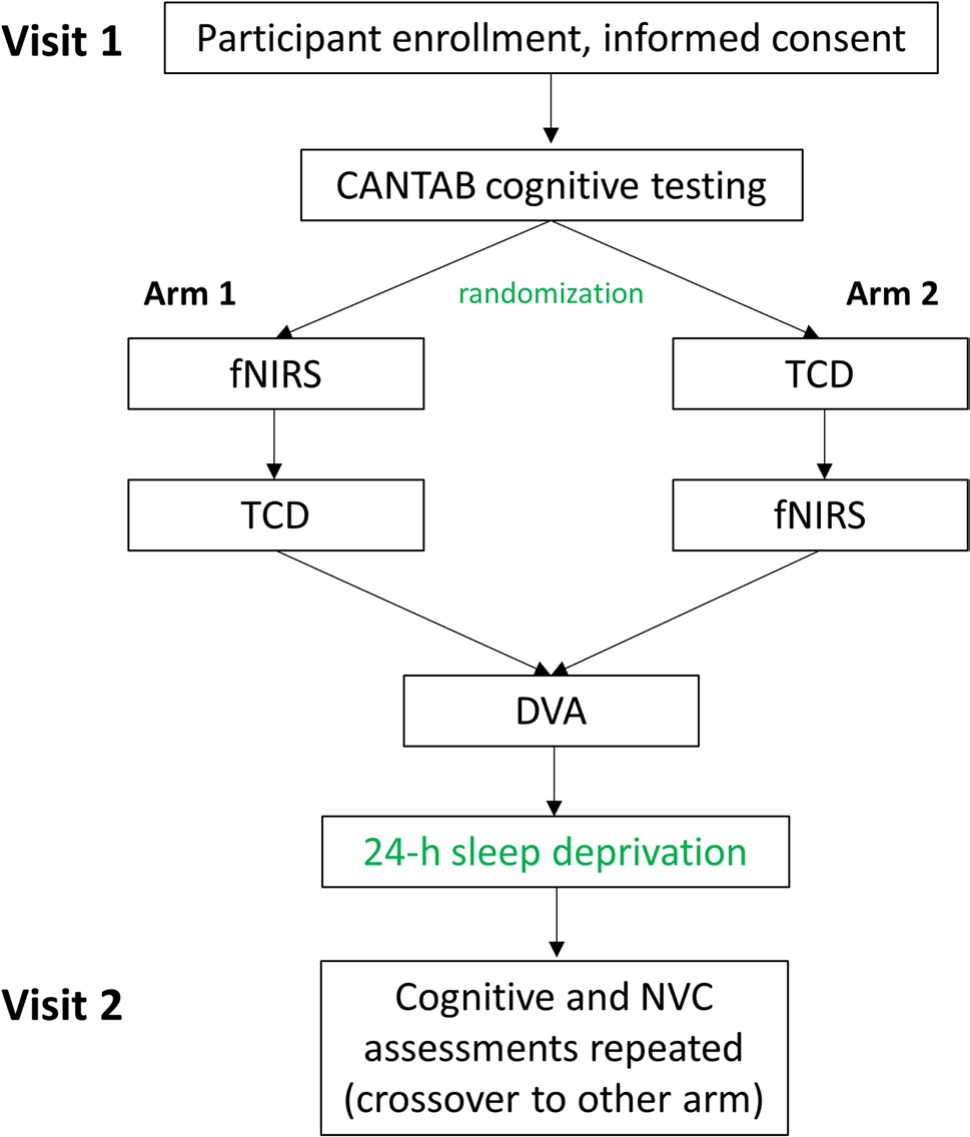
Flowchart showing the study protocol. CANTAB: Cambridge Automated Neuropsychological Test Automated Battery, TCD: Transcranial Doppler sonography, fNIRS: functional Near-Infrared Spectroscopy, DVA: Dynamic retinal Vessel Analysis.

Participants were asked to refrain from consumption of caffeinated beverages for at least 6 h before baseline and 24 h before experimental day assessments. Blood pressure was measured before and after sleep deprivation. Participants were randomized to start with transcranial Doppler sonography (TCD) or functional near infrared spectroscopy (fNIRS) assessments during the NVC assessment block of the baseline visit and were crossed over at the experimental day visit. The dynamic retinal vessel analysis (DVA) examination concluded both visits (a flowchart of the protocol is shown in Fig. 1).

### Cognitive assessments

Cognitive testing was performed using the cloud-based, computerized Cambridge Neuropsychological Test Automated Battery (CANTAB, Cambridge Cognition, Cambridge, UK)^21^ as previously described^22^. Briefly, the testing started with the Motor Screening Task to detect any sensorimotor deficit. Once the Motor Screening Task was successfully completed, participants were presented with remaining tests to evaluate several cognitive domains, including sustained attention and psychomotor speed (Rapid Visual Processing, RVP test and Reaction Time, RTI tests), spatial working memory (SWM test), visual memory, short-term recognition, and visual information matching (Paired Associates Learning, PAL and Delayed Matching to visual Sample, DMS tests). All participants started with the computerized cognitive testing. All tests were done uninterrupted, in a quiet environment. To minimize learning effects, CANTAB automatically creates parallel versions of tests^21^.

In addition, an *n*-back approach-based cognitive test was used to evoke NVC responses during TCD assessment and results of the *n*-back test were also analyzed for cognitive performance. The *n*-back test is designed to assess working memory, as previously described^11,17^. Briefly, after explanation of the assessment, participants were seated in front of a 22-inch monitor, with their dominant hand on the computer mouse. Participants were tested using the following sequence of *n*-back tasks: 1) 0-back (click the mouse button every time the letter “W” is presented), 2) 1-back (click the mouse button every time any letter repeats itself in a sequence, e.g. x–y-A-A), 3) 0-back (see above), 4) 2-back (click mouse button every time any letter repeats itself 2 letters back, e.g. x-A-y-A). Each task consisted of the presentation of 60 letters (each appearing for 250 ms), and the period between letters was randomized (minimum 1850 ms, maximum 2050 ms). For each task, reaction time and response accuracy were recorded, latter expressed as the percentage of correct answers. To minimize practice effects, a different sequence of letters were presented when the task was administered on the second visit.

### Cerebral blood flow (CBF) and assessment of neurovascular coupling-related hemodynamic responses using transcranial Doppler (TCD) sonography

The mean blood flow velocity in the middle cerebral artery (MCAv) was measured using TCD sonography (Digi-Lite, Rimed, Raanana, Israel) bilaterally using two 2-MHz ultrasound probes positioned over the temporal acoustic windows. Probes were mounted on a fixation device that allowed monitoring of MCAv at a constant angle for extended periods of time. Left and right MCAv signals were identified according to the standardized criteria guided by signal depth and velocity^23,24^. After establishing optimal MCAv signal, probes were secured, and the signal depth and power remained constant throughout the test session. Channels with inadequate temporal acoustic window were excluded from the analysis.

To evoke NVC responses during TCD recording, cognitive stimulation with the *n*-back approach (see above) was used. Each *n*-back task was approximately 130 s long, and MCAv was averaged for a 100 s period, starting 10 s after the task has started to eliminate the effect of motion and breathing-related artefacts at the beginning of the administered task. Evoked NVC was evaluated by the following equation using the 100 s MCAv average values: [*n*-back] / [preceding 0-back].

### Assessment of neuronal activity-evoked hemodynamic responses using functional near infrared spectroscopy (fNIRS)

**Figure 2:**
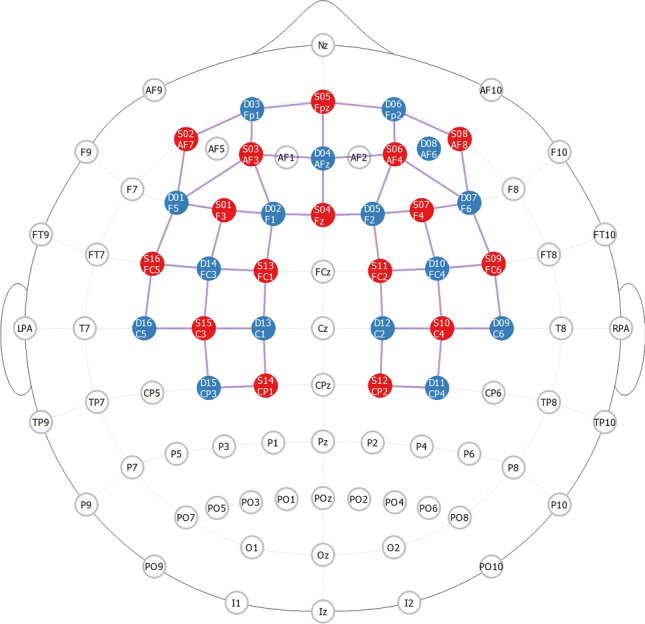
Optode placement during functional Near-Infrared Spectroscopy (fNIRS) assessments. fNIRS probe design was based on optode positioning in the international 10–20 space, and optodes were mounted in a modified electroencephalography cap in the corresponding position. Red dots represent light source, blue dots represent light detector optodes. Probe channels are shown as purple lines. The fNIRS probe and current figure was created with NIRSite 2020.7 (www.nirx.net).

Functional NIRS examinations were performed utilizing the NIRScout platform (NIRx Medical Technologies LLC, NY, USA) equipped with 16 light sources and 16 detector optodes. A128-port headcap (Easycap GmbH, Woerthsee-Etterschlag, Germany) was positioned over the head to cover the area of the international 10–20 space (as shown in Fig. 2). The line between Fpz and Iz ports on the headcap was aligned with the sagittal plane of the head, and the optode in the Fpz position of the cap was aligned with Fpz on the subject. The cap was set up with spacers that limit the variability of distance between optodes to an average source-detector distance of 3 cm. Placement of the optodes covered the prefrontal cortex, dorsolateral prefrontal cortex, and also included the medial motor and somatosensory cortex. Sufficient coverage of these regions was determined by projection of channel position to the cortical surface within the Montreal Neurological Institute (MNI) coordinate space (Supplemental Table S1)^25,26^. Measurements were performed in a quiet and darkened room.

To evoke NVC responses during fNIRS recording, a finger tapping task was used. Participants were seated with both hands rested on a desk in front of them. A built-in finger tapping stimulation paradigm was administered using the NIRStim software (NIRx Medical Technologies LLC, NY, USA). Participants were presented with auditory commands to start tapping either their left or right index finger against the surface of the desk. Three finger tapping sessions were administered alternating left and right finger tapping. Participants were unaware of the stimulation order prior to the start of the examination. Each left and right hand finger tapping had a duration of 10 s and an interval of 10 s between tapping sessions.

### Analysis of fNIRS data

Functional NIRS data were analyzed using a pipeline based on General Linear Model (GLM) approach created using the Brain AnalyzIR toolbox (commit 2d7beed)^27^. Briefly, measured optical densities were converted to change of hemoglobin concentration using the Beer-Lambert law^28^, pre-whitening of data was performed with an autoregressive model based algorithm^29^, and a discrete cosine transform based high-pass filter (0.08 Hz) was used to remove slow drift. The design matrix included six boxcar regressors (one for each finger-tapping trial), which were convolved with a canonical hemodynamic response function to predict brain activation. Parameter estimates (beta-weights), scaling the predictors, were then used for group level statistics. Group level statistics were performed using a mixed effects model. The model was defined in a Wilkinson-Rogers formula^30^ of ‘beta ~ -1 + SD state + (1|Subject)’. Output of the mixed effects model statistics were used for a t-test, where a t-contrast of hemodynamic responses of [+ 1, -1] or [pre-SD – post-SD] was applied. Increased activation was considered significant where false discovery rate corrected *p* < 0.05.

### Neurovascular coupling-related microvascular response assessment using dynamic retinal vessel analysis (DVA)

Dynamic retinal vessel analysis was performed as previously described^20^. Briefly, all participants were examined for visual acuity using Snellen chart in a well-lit room, ocular pathologies using a slit-lamp, and evaluated for intraocular pressure using a Goldmann applanation tonometer. All measurements were performed on the right eye.

For DVA, the pupil of the right eye was dilated using topical application of tropicamide (1% Tropicamide Ophtalmic Solution USP, AKORN, Lake Forest, IL), followed by a period of rest to achieve stable hemodynamic conditions. Retinal arteriolar and venular diameters were evaluated using the Dynamic Vessel Analyzer (DVA, IMEDOS, Jena, Germany) according to published protocols^20,31,32^. NVC responses in retinal vessels were evaluated in response to flicker light stimulation^33^. The flicker frequency was set to 12.5 Hz and a duration of 20 s. Before starting the flicker stimulation, a baseline recording for a minimum period of ~ 100 s was performed. The statistical mean of 3 consecutive examinations was calculated for each participant and each evaluated parameter. Vessel segments of approximately 1 mm in length, located in the upper temporal quadrant 1–3 optic disc diameters away from the optic disc edge, were assessed in the arteriolar and venular branches^33^. Sites where two vessels were very close to each other were avoided.

### Statistical analysis

The effect of sleep deprivation on cognitive function and NVC-related hemodynamic responses measured using TCD and DVA was evaluated using non-parametric Wilcoxon matched pairs signed rank test or parametric paired t-test after testing the dataset for normality. In the case of normally distributed data and non-normally distributed data, the mean ± SD and median [interquartile range] were reported respectively. The difference of p < 0.05 was considered statistically significant. These data were analysed and presented in table formats using Jamovi and graphically presented using GraphPad Prism Version 8 (GraphPad Software, San Diego, CA USA).

## Results

### Study participants

According to the 7-day sleep log of participants, on average, all subjects slept 7.9 ± 0.6 h every night, fell asleep within 11.3 ± 9.6 min, woke up 0.7 ± 0.6 times per night, experienced 0.04 ± 0.07 times of stress daily, consumed 0.6 ± 0.6 coffee or alcoholic beverages daily, and reported sleepiness of 3.1 ± 0.9 on a scale from 1 to 10 (from non-sleepy to very sleepy).

Blood pressure recordings averaged to 119 ± 6 mmHg systolic pressure and 80 ± 6 diastolic pressure pre-SD and 116 ± 8 mmHg systolic pressure and 71 ± 12 diastolic pressure post-SD. There was no statistically significant difference in arterial blood pressure measurements before and after sleep deprivation.

### Sleep deprivation impairs reaction time and sustained attention

**Figure 3:**
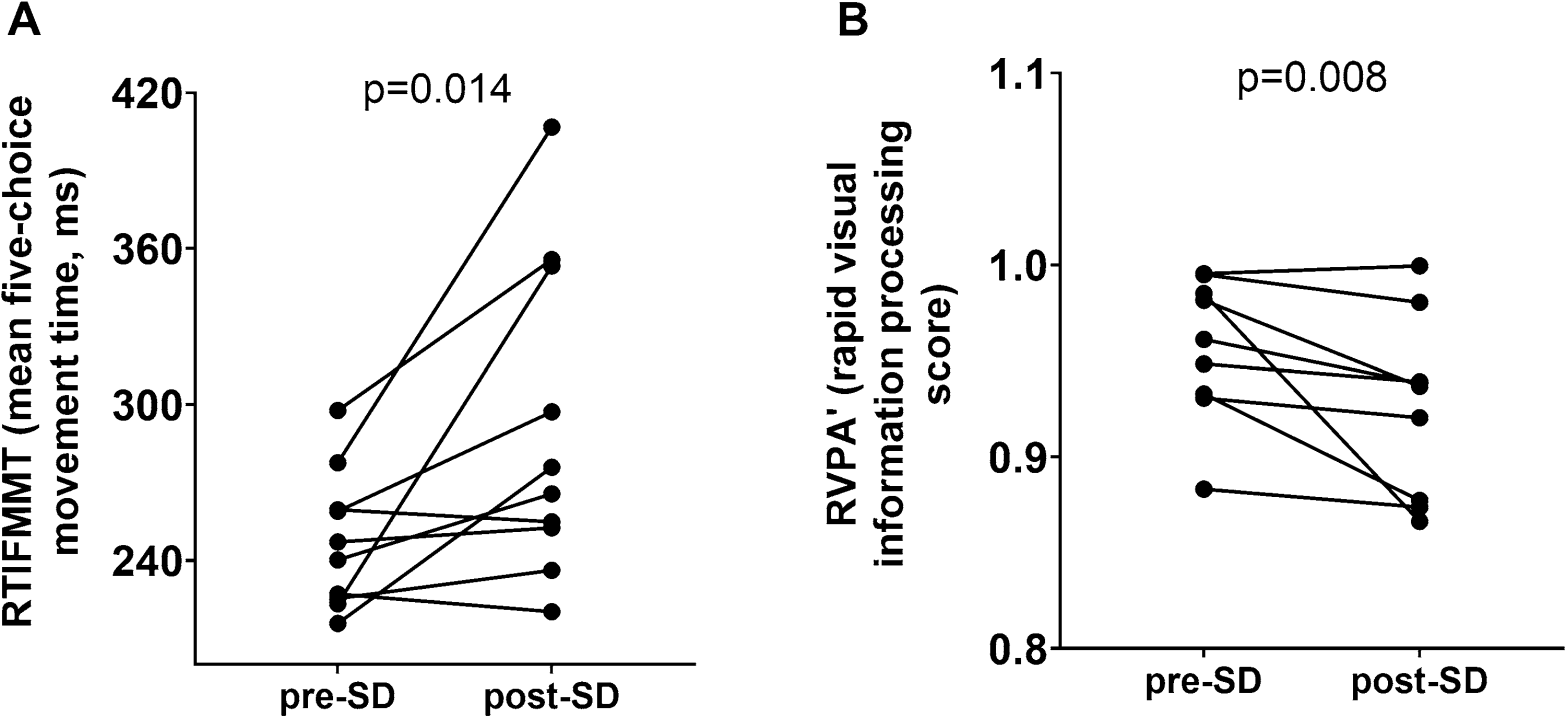
Sleep deprivation impairs cognitive performance. Cognitive performance was evaluated before and after 24 h of sleep deprivation in 10 healthy young (27.6 ± 3.7 years of age) male individuals using a CANTAB battery of tests. Reaction time was measured with the RTI test, and we observed a significant increase in reaction time after sleep deprivation (panel A). Sustained attention was assessed with the rapid visual information processing test (RVP), and we found a significant decrease in performance in this cognitive domain (panel B). Wilcoxon signed-rank test was used for comparisons. p < 0.05 was considered significant. RTIFMMT: Reaction time mean five choice movement time. RVPA: Rapid Visual Processing A’ (A prime) a signal detection measure of the subject’s sensitivity to the target sequence of three numbers, a metric of how good the subject was at detecting targets. Maximal value is 1, being the highest score for the best performance.

Results of the CANTAB assessment are presented in Supplemental Tables S2–S4. Out of six tests performed, sleep deprivation significantly impaired reaction time (Fig. 3A), evidenced by a significant increase in movement time during the five-choice reaction time test (RTIFMMT: The mean time taken for a subject to release the “standby” button and select the target button, ms).

Sleep deprivation has also significantly impaired sustained attention (Fig. 3B), evidenced by a decrease in ability of the participant to recognize and identify three different strings of three numbers in a sequence of changing numbers (RVPA: Rapid Visual Processing A’), a score describing the performance of the subject in detecting either a string of numbers in a sequence of running numbers, scale 0 to 1, 1 being the highest score.

No other cognitive domain examined by the CANTAB tests (spatial working memory, visual memory, short-term recognition, visual information matching) was affected by the short-term sleep deprivation applied in current study. Working memory performance was also tested using *n*-back cognitive test. N-back test found no significant difference in the percentage of correct responses (1-back: 98.33 [98.33 to 100]% pre-SD vs. 98.33 [96.67 to 100]% post-SD, p = 0.66; 2-back: 98.33 [95.83 to 100]% pre-SD vs. 96.67 [90.83 to 100]% post-SD p = 0.09), and reaction time was significantly different during the 1-back condition (502 ± 140 ms pre-SD vs. 542 ± 146 ms post-SD, p < 0.01), but not the 2-back condition (657 ± 184 ms pre-SD vs. 665 ± 201 ms post-SD, p = 0.83).

### Sleep deprivation alters cerebral blood flow (CBF) and NVC-related hemodynamic responses in the brain cortex

**Figure 4:**
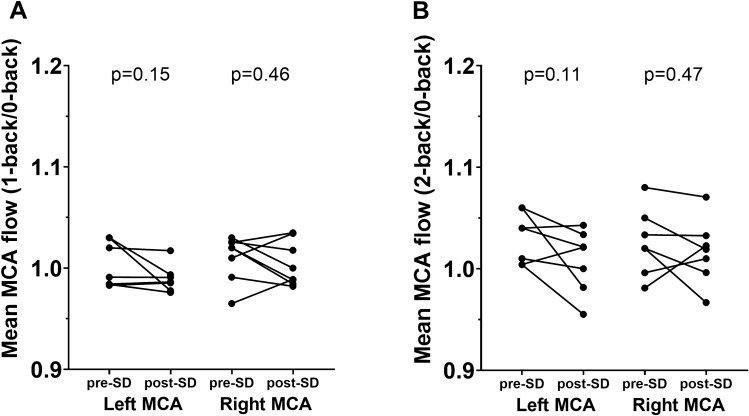
The effect of sleep deprivation on NVC-related hemodynamic responses measured in the middle cerebral artery using transcranial Doppler sonography. Hemodynamic NVC responses were measured in both middle cerebral arteries (MCA) in response to cognitive *n*-back test using transcranial Doppler sonography. NVC responses were evaluated during 1-back and 2-back tasks, normalized to preceding 0-back task. We observed a non-significant trend for a decrease in NVC in the left MCA during both 1-back (panel A) and 2-back tasks (panel B). Wilcoxon signed-rank test was used for comparisons. p < 0.05 was considered significant.

To evaluate the effect of sleep deprivation on NVC-related hemodynamic responses in the brain, we measured changes in CBF velocities in left and right middle cerebral arteries (MCAv) using TCD in response to cognitive stimulation with *n*-back test. One subject was excluded from TCD analysis due to inadequate signal over both left and right temporal acoustic windows. One-sided MCAv data were excluded for 1 subject due to inadequate signal on the left side and for 2 subjects due to inadequate signal on the right side. MCAv measured during 2-back was excluded for one subject due to deviation from the task. TCD did not detect significant difference when comparing baseline MCAv between pre-SD and post-SD timepoints. On the contrary, when the cognitive *n*-back test was administered, right MCAv was markedly slower after SD (Table 1). Interestingly, when MCAv during *n*-back was normalized to preceding 0-back task, we did not observe a significant change in relative blood flow velocity (Fig. 4).

**Table 1:** Middle cerebral artery (MCA) blood flow velocity before and after 24 h of sleep deprivation.

	Condition	PRE-SD	Post-SD	*p*
Left MCAv	Baseline	47.3 ± 1.2	47.5 ± 1.7	0.87
	0-back #1	45.8 ± 1.5	44.8 ± 1.7	0.57
	1-back	46.1 ± 1.4	44.1 ± 2.0	0.33
	0-back #2	44.4 ± 1.5	43.8 ± 1.6	0.76
	2-back	45.7 ± 1.5	44.1 ± 1.6	0.53
Right MCAv	baseline	44.4 ± 1.7	41.7 ± 1.5	0.18
	0-back #1	44.5 ± 1.6	40.0 ± 0.4	0.02*
	1-back	44.9 ± 1.4	40.2 ± 0.6	0.01*
	0-back #2	43.1 ± 1.4	39.3 ± 0.7	0.04*
	2-back	44.5 ± 1.6	40.1 ± 0.9	0.04*

Blood flow in the middle cerebral artery (MCAv) was measured by transcranial Doppler sonography (TCD) before and during *n*-back cognitive stimulation in healthy young adults pre- and post 24 h of sleep deprivation (SD). A paired *t*-test was used to compare observed MCAv.

*p < 0.05.

**Figure 5:**
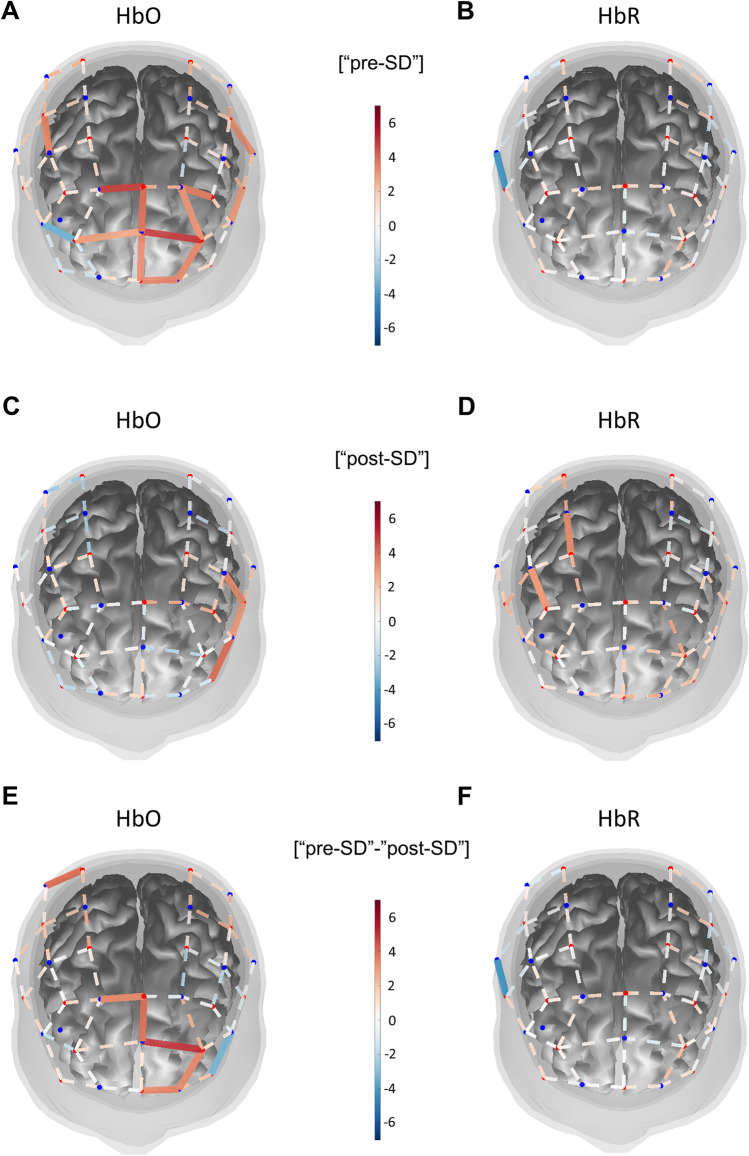
Sleep deprivation significantly impairs cortical hemodynamic responses measured using functional near-infrared spectroscopy. During functional near infrared spectroscopy (fNIRS) assessments, NVC-related hemodynamic responses were evoked by performing a finger tapping task initiated by auditory commands. Participants were seated in front of a desk, and an fNIRS cap covering areas of the prefrontal cortex and motor cortex was positioned on their head. A stimulation software instructed subjects with an auditory command to tap with the left or right index finger. Three 10 s trials were recorded for both hands. A General Linear Model based approach was used to extract beta weights estimating hemodynamic signal. Parameter estimates were used for further group-level statistics. Group level statistics were performed using mixed effect model statistics. Group average of responses are shown pre-SD (oxyhemoglobin, HbO: Panel A, deoxy-hemoglobin, HbR: Panel B) and post-SD (HbO: Panel C, HbR: Panel D). A *t*-contrast of [+ 1, −1], [pre-SD-post-SD] was applied on group level results. NVC responses were more pronounced: HbO was increased (Panel E) and HbR was decreased (Panel F) pre-SD when comparing it to signals recorded post-SD. The Brain AnalyzIR Toolbox^27^ was used to generate result images. Results of the group level statistics are plotted as a 3D mesh over the Colin27 atlas^73^. Red and blue dots represent the optodes of the probe (red: light source, blue: detector). Solid lines connecting optode positions represent channels with statistical significance (FDR corrected *p* < 0.05), plotted values are *t*-values obtained through a *t*-test, warm colors representing *t*-values greater than 0, cold colors representing *t*-values lower than 0. Numerical results are shown in Supplemental Table S5–S6.

Further, we evaluated the effect of sleep deprivation on NVC-related hemodynamic responses by measuring hemodynamic changes (oxy- and deoxy-hemoglobin, HbO and HbR) in the brain cortex in response to finger tapping task using the fNIRS approach. One subject was excluded from the analysis due to high variability noise in the NIRS signal in > 80% of channels. Results of the GLM based approach are presented in Fig. 5 and Supplemental Table S5, S6. When evaluating hemodynamic changes recorded during the finger tapping task, we observed diminished responses post-SD (Fig. 5C,D) when compared to the pre-SD state (Panel 5A-B). A *t*-contrast between “pre-SD” and “post-SD” was used. Higher HbO and lower HbR, representing a more pronounced hemodynamic response, were found in brains pre-SD in a widespread area of the prefrontal cortex, including the frontopolar cortex. SD decreased HbO and increased HbR signal amplitudes in the triangular area of the left inferior frontal gyrus. Increased HbO signal was also found in the area of the right primary somatosensory cortex pre-SD (Panel 5E-F).

**Figure 6:**
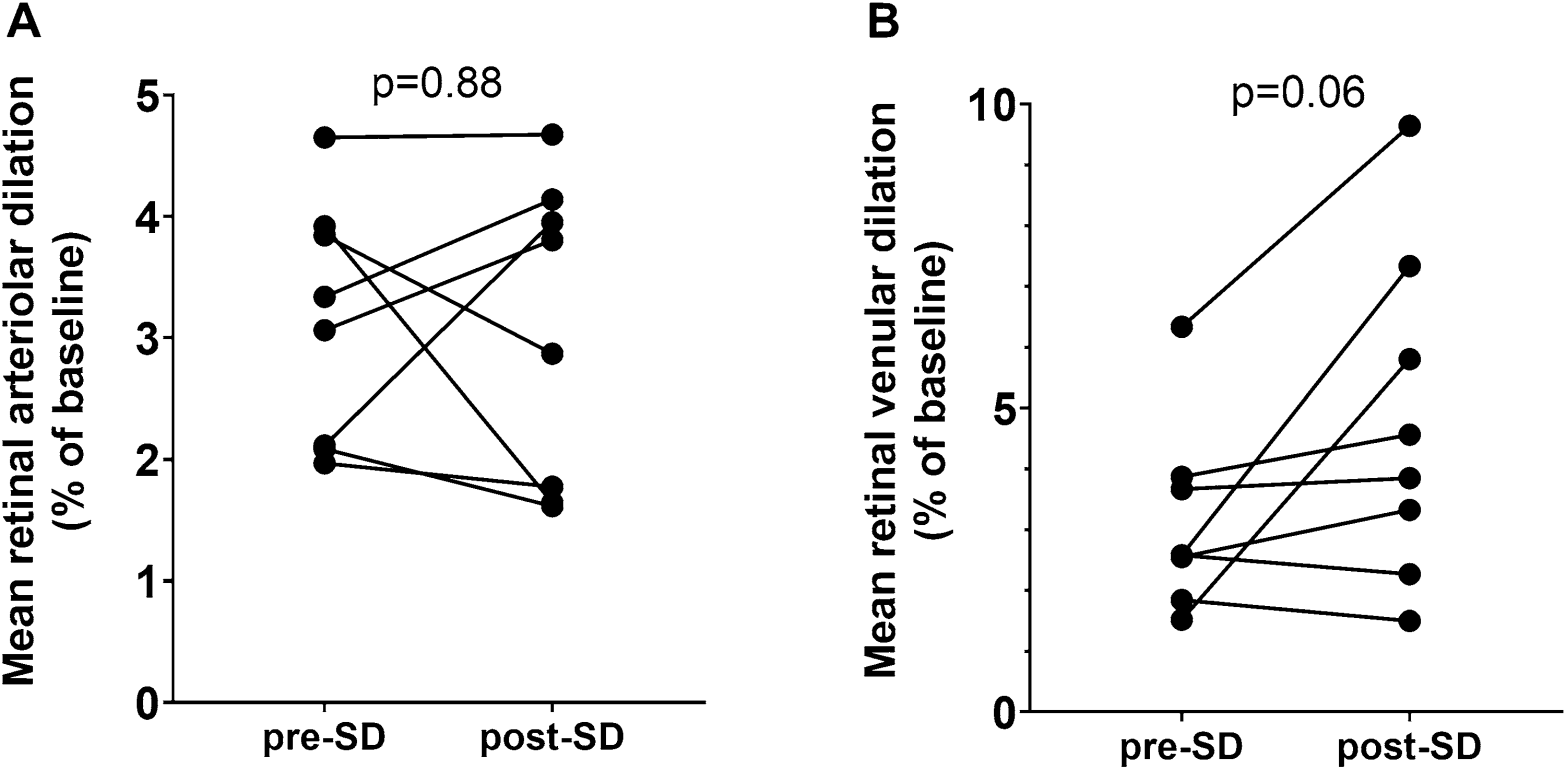
The effect of sleep deprivation on NVC-related hemodynamic responses measured in retinal arterioles and venules in responses to flicker light photoreceptor stimulation. Flicker light photoreceptor stimulation was used during Dynamic retinal Vessel Analysis (DVA) assessments to evoke NVC in retinal vessels. No statistically significant effect of sleep deprivation was observed in evoked retinal arteriolar dilation (panel A). Note the SD tends to alter flicker light-induced retinal venular dilation (panel B). Paired *t*-test was used for comparisons. p < 0.05 was considered significant.

Finally, the effect of sleep deprivation in NVC was measured in retinal vessels in response to flicker light stimulation. Out of 10 participants, 8 were able to fully complete assessments before and after sleep deprivation. Two participants reported major difficulties to focus on the target during assessment following sleep deprivation and were excluded from analysis. Although SD did not cause statistically significant alterations in the measured retinal arteriolar NVC responses, retinal venular dilation tended to increase (*p* = 0.06) after 24 h of SD (Fig. 6).

## Discussion

The present study provides evidence that short-term SD promotes cognitive deficits and alters CBF and cortical NVC-related hemodynamic responses in the brain.

Significant impairment was found in the domain of sustained attention, and reaction time was also slower after 24 h of SD. Working memory accuracy was found intact according to the results of the *n*-back test, however, the test used for sustained attention (Rapid Visual Information Processing test) also probes working memory^10^, during which impaired accuracy was detected. No significant effect of short-term SD on visual memory, short-term recognition and visual information matching was observed. Cognitive testing results obtained in our study accord with findings of previous studies. A study done on healthy young individuals showed that one night of sleep deprivation leads to an increase in reaction times without higher error rate during a Stroop color-naming task^34^. Another recent study investigated the association between subjective sleep quality and cognitive performance, and found no correlation between sleep quality and cognitive performance in domains of working memory and executive function^35^. On the contrary, a study utilizing similar methodology detected changes in working memory after 24 h of SD with the Rapid Visual Information Processing test and an *n*-back test, although the *n*-back test also contained a more difficult 3-back task^10^. Earlier studies also report that a state of continuous wakefulness for over 16 h is significantly associated with a decrease in reaction time and worsening of performance accuracy on tests of psychomotor vigilance^36^. In fact, there is a growing body of evidence suggesting that even 4–6 h of sleep restriction can induce a significant slowing of response times on cognitive tasks. If this sleep restriction is chronically prolonged to a period of 2 weeks, the deficit in reaction time can reach the magnitude of deficit observed in individuals with over 48 h of sleep deprivation^37,38^. Previous clinical studies demonstrate that sustained attention is gradually impaired over the course of sleep restriction^39,40^and small differences in sustained attention that are observed prior to sleep deprivation are significantly amplified during the period of prolonged wakefulness^39^.

Ample studies have addressed mechanisms of cognitive deficits during SD. Clinical studies that utilized neuroimaging techniques such as positron emission tomography demonstrated a significant reduction in metabolic activity in sleep deprived individuals when measured in brain regions responsible for attention, information processing and executive function, alertness and cognitive performance^41^. Metabolic activity of the brain is directly linked to hemodynamic adjustments to provide active neurons with oxygen and other nutrients^15^. An earlier study, which used functional magnetic resonance imaging (fMRI) to detect blood oxygen level dependent (BOLD) signal in the brain of sleep deprived individuals during the psychomotor vigilance task, mainly focused on the neural basis and concluded that optimal performance on the cognitive task relied on activation of brain regions responsible for sustained attention and motor systems^42^. The majority of other studies that utilized the fMRI approach to evaluate the association between hemodynamic changes during SD and cognitive function found significant changes in the brain regions responsible for executive function. However, it should be noted that the aforementioned studies were done primarily in resting state^43^, whereas our study examined NVC during neuronal activation to different stimuli.

Growing evidence suggests that NVC impairment is causally related to cognitive dysfunction in a spectrum of pathophysiological conditions^14,15^. In the present study, we examined the impact of short-term SD on NVC responses using a range of external stimuli, including cognitive *n-*back test, finger tapping motor task, and flicker light photoreceptor stimulation. We found that short-term SD alters hemodynamic NVC responses in multiple cortical areas measured with fNIRS during finger tapping task. Specifically, a significantly smaller task-related hemodynamic response was seen post-SD, represented by decreased HbO and increased HbR signals in a widespread area of the prefrontal cortex. Higher pre-SD HbO signal was also seen in an isolated area of the somatosensory cortex. All of these changes indicate impairment of NVC-related hemodynamic responses post-SD, however, further investigation is necessary to reveal if this impairment is a consequence of the dysfunction of supplying blood vessels, neuronal activation, or neurovascular coupling itself.

The impact of sleep deprivation on NVC-related relative increase in blood flow was not significant, when measured in the middle cerebral artery (MCA) using TCD. Interestingly, post-SD, we observed a unilateral decrease in absolute MCA blood flow velocity during the *n*-back cognitive stimulation. NVC responses assessed directly in retinal arterioles and venules in response to flicker light stimulation also did not yield significant results, demonstrating only a trend for increased venular dilation, suggesting that there may be minor disturbances in cerebrovascular regulation. Together, these findings suggest that SD is associated with impairments in cortical NVC-related hemodynamic responses and altered CBF. Following recent preclinical studies that demonstrated that SD impairs NVC responses^44^ and that experimentally induced neurovascular uncoupling results in impaired cognitive performance, it is possible that SD itself contributes to NVC impairment^14,15,45^.

NVC involves a complex interaction among activated neurons, astrocytes and microvascular endothelial and smooth muscle cells. The mechanisms underlying SD-induced impairment of NVC responses are not well understood and may include changes in neuronal activation, dysfunction of astrocytes and/or altered production of vasodilator mediators by microvascular endothelial cells. Our results indicate a widespread decrease of hemodynamic NVC responses over the prefrontal cortex during a motor task with auditory cues. Although we did not detect changes in the motor cortex while asking our subjects to perform a motor task, this simple motor task also required participants to cooperate, pay attention, and execute the given instructions. Consistent with our data, it has been described that lesions of the frontopolar cortex do not cause widespread cognitive decline, but only deficits on a restricted set of tasks^46^. A meta-analysis investigating the effects of prefrontal cortex lesions on cognitive function found that patients with frontopolar cortex lesions presented with lower performance in sustained attention tasks^47^. Astrocyte activation also plays a key role both in NVC and modulation of neuronal function (e.g. regulation of energy metabolism and synaptic transmission), yet, its contribution to SD-induced changes in functional hyperemia and/or cognitive function remains elusive. It has been shown that prolonged wakefulness leads to accumulated sleep pressure (the need for sleep), which may cause impairment of memory and learning in human subjects^48^. Interestingly, preclinical studies show that astrocytes modulate sleep pressure via purinergic transmission, and that modulation of this pathway has a direct effect on cognitive outcomes^49^.

Release of vasodilator nitric oxide (NO) from microvascular endothelial cells also plays an important role in NVC. Neurovascular dysfunction associated with microvascular endothelial dysfunction was shown to contribute to the pathogenesis of age-related cognitive decline^50–55^. There is evidence suggesting that short-term SD is associated with endothelial dysfunction both in experimental animals and in human subjects^56^. A study based on a similar cohort of 12 healthy young men, averaged 29 years of age, showed that total SD of over 29 h may cause generalized microvascular dysfunction, including impairment of endothelial regulation of cutaneous microvascular blood flow and increased circulating biomarkers of endothelial dysfunction^56^. Preclinical studies also demonstrate that SD in rats results in marked endothelial dysfunction associated with nitric oxide synthase and cyclooxygenase pathway alterations^57^ and persisting alterations in CBF^44^. We have also demonstrated alterations in CBF while participants were performing a cognitive task, however, TCD could not detect differences in baseline CBF after 24 h of SD. These findings suggest that sleep deprivation results in CBF dysregulation and decreased compliance that could, potentially, limit further dilation of blood vessels, thus limiting blood delivery and altering NVC responses. In the present study, short-term SD did not significantly affect NVC responses evoked by flicker light stimulation in the retinal arterioles, which supports the concept that short-term SD does not cause generalized endothelial or microvascular dysfunction. On the basis of this finding, we propose that region-specific alterations in the function of the neurovascular unit caused by SD should be considered in future studies.

The present study has several limitations. Due to quality control measures, some of the data were excluded from all three methods examining the hemodynamic components of NVC, partly due to difficulties caused by sleep deprivation itself. The reduction in observation numbers may have impacted the statistical power of TCD or DVA assessments. In that regard it should be also noted that the fNIRS setup used in current study utilized 48 channels that provided additional sensitivity for detecting changes in cerebral circulation as compared to TCD and DVA methods. In addition to small sample size, our study did not allow us to investigate the effect of sleep deprivation on other components involved in NVC, such as neuronal activity and astrocytic function. Simultaneous assessment of hemodynamic responses with fNIRS and electrical activity with EEG during cognitive/motor stimulation may provide additional information for the neuronal contribution to NVC responses. Future studies with pharmacological agents such as indomethacin, to inhibit astrocytic function mediated via cyclooxygenase pathway, may also provide additional insights into the mechanisms of NVC impairment induced by SD. Further limitation of the study is the inability to explore sex effects, as only men were enrolled.

Current study also addressed methodological questions. Cognitive domains of working memory, spatial working memory and executive function that are known to decline with neurodegeneration with or without vascular pathology^58^. Spatial working memory and executive function were not affected by sleep deprivation, however, our results indicate that working memory may be affected by SD. In the current study, *n*-back testing did not reveal any significant change in accuracy, however, the performed RVP test returned the result of decreased accuracy post-SD. Apart from sustained attention, RVP test also probes working memory. Other studies reported that the effect of SD on working memory performance also depended on other factors, such as the age of studied population^59^, or the difficulty of the task^8,59^. These together suggest that studies focusing on the neurodegeneration and cognitive impairment need to use caution when finding isolated impairments of working memory. Our results also suggest that fNIRS methodology is more sensitive to the effects of sleep deprivation, and this has to be kept in mind when interpreting fNIRS results. Recently, fNIRS methodology emerged as an alternative for brain-computer interface^60^, and our results suggest that the magnitude of fNIRS signals recorded over certain cortical areas may be different even after short-term SD.

It should also be noted that our study participants were young and healthy individuals without any known comorbid condition, which may alter NVC responses. Heightened state of inflammation associates with increased levels of inflammatory cytokines, including Tumor Necrosis Factor-α (TNF-α) and interleukin-1 (IL-1) which both alter blood flow and NVC, and their role in sleep regulation was also described^44,61^. Therefore, it is likely that older adults, especially those affected by cardiovascular risk factors (including hypertension, diabetes mellitus, obesity, etc.) or brain pathologies (e.g. subclinical Alzheimer's diseases^62^ and other pathological conditions) associated with a heightened inflammatory state may be more susceptible to the adverse effects of SD, exacerbating both neurovascular dysfunction and cognitive impairment. In fact, aging pathological conditions that promote accelerated aging were shown to induce significant changes in the phenotype and function of neurovascular unit^63–69^, which are expected to impact both NVC responses and cognitive function^68,69^. It is also likely that chronic sleep deficits may have more pronounced effects both on NVC and cognitive function than short-term SD. Considering that prevalence of chronic insomnia is highest (20%) in adults age 65 and over^70^, further studies are needed to investigate the neurovascular and cognitive consequences of chronic SD in older adults.

## Conclusion

Collectively, our results demonstrate that 24-h sleep deprivation significantly impairs sustained attention and reaction time, without profound changes in cognitive domains of working memory, visual memory, and short-term visual information matching. Following sleep deprivation, altered cerebral blood flow was detected by TCD and decreased NVC-related hemodynamic responses were detected by fNIRS in the prefrontal cortex, providing a possible explanation for these deficits.

## Supplementary Information

Supplementary Tables.

## Data Availability

Raw NIRS data that were used for this study are also available in the following Physionet^71^ database: https://doi.org/10.13026/669h-cx11^72^.
