# Supplementary material for: Sleep deprivation impairs cognitive performance, alters task-associated cerebral blood flow and decreases cortical neurovascular coupling-related hemodynamic responses
Source: Sci Rep. 2021 Oct 25;11:20994. doi: 10.1038/s41598-021-00188-8 (PMC8546061; doi:10.1038/s41598-021-00188-8)
Supplement: Supplementary file 1 — Supplementary Tables. [file 41598_2021_188_MOESM1_ESM.pdf]

SUPPLEMENTAL MATERIAL FOR THE MANUSCRIPT

"Sleep deprivation impairs cognitive performance, alters task-associated cerebral blood flow and decreases cortical neurovascular coupling-related hemodynamic responses"

Tamas Csipo[1,2,3,#], Agnes Lipecz[1,3,4,#], Cameron Owens[1,5], Peter Mukli[1,3,6] , Jonathan W. Perry[1], Stefano Tarantini[1], Priya Balasubramanian[1], Ádám Nyúl-Tóth[1], Valeriya Yabluchanska[1], Farzaneh A. Sorond[7], J. Mikhail Kellawan[5], György Purebl[8], William E. Sonntag[1], Anna Csiszar[1,9], Zoltan Ungvari[1,9,10,11], Andriy Yabluchanskiy[1,11,*]

[1]Vascular Cognitive Impairment and Neurodegeneration Program, Oklahoma Center for Geroscience/Reynolds Oklahoma Center of Aging, Department of Biochemistry and Molecular Biology, University of Oklahoma Health Sciences Center, Oklahoma City, OK, USA

[2]Department of Cardiology, Division of Clinical Physiology, Faculty of Medicine, University of Debrecen, Debrecen, Hungary

[3]International Training Program in Geroscience, Doctoral School of Basic and Translational Medicine/Department of Public Health, Semmelweis University, Budapest, Hungary

[4]Department of Ophthalmology, Josa Andras Hospital, Nyiregyhaza, Hungary

[5]Department of Health and Exercise Science, University of Oklahoma, Norman, OK, USA

[6] International Training Program in Geroscience, Department of Physiology, Faculty of Medicine, Semmelweis University, Budapest, Hungary

[7]Department of Neurology, Division of Stroke and Neurocritical Care, Northwestern University Feinberg School of Medicine, Chicago, IL, USA

[8]Institute of Behavioral Sciences, Semmelweis University, Budapest, Hungary

[9]International Training Program in Geroscience, Theoretical Medicine Doctoral School/Departments of Cell Biology and Molecular Medicine and Medical Physics and Informatics, University of Szeged, Szeged, Hungary

[10]International Training Program in Geroscience, Doctoral School of Basic and Translational Medicine/Department of Public Health, Semmelweis University, Budapest, Hungary

[11]Department of Health Promotion Sciences, College of Public Health, University of Oklahoma Health Sciences Center, Oklahoma City, OK

[#]These authors contributed equally to this work

*Corresponding author

Supplemental Table 1. Cortical projection of channels used during functional near-infrared spectroscopy (fNIRS) assessments.

source	detector	channel coordinates (MNI)			label
		X	Y	Z	
**1**	**1**	-41	43	24	Frontal_Mid_L
**1**	**2**	-24	40	39	Frontal_Sup_L
**1**	**14**	-42	24	33	Frontal_Mid_L
**2**	**1**	-28	33	4	Frontal_Inf_Tri_L
**2**	**3**	-27	56	2	Frontal_Sup_L
**3**	**1**	-32	50	17	Frontal_Mid_L
**3**	**2**	-16	51	33	Frontal_Mid_L
**3**	**3**	-21	61	6	Frontal_Sup_L
**3**	**4**	-9	61	28	Frontal_Sup_L
**4**	**2**	-7	42	50	Frontal_Sup_L
**4**	**4**	5	39	31	Frontal_Sup_Medial_L
**4**	**5**	13	48	53	Frontal_Sup_R
**5**	**3**	-12	72	1	Frontal_Med_Orb_L
**5**	**4**	3	63	17	Frontal_Sup_Medial_L
**5**	**6**	15	70	0	Frontal_Med_Orb_R
**6**	**4**	21	61	26	Frontal_Sup_R
**6**	**5**	24	48	27	Frontal_Mid_R
**6**	**6**	28	47	6	Frontal_Mid_R
**6**	**7**	41	39	9	Frontal_Inf_Tri_R
**7**	**5**	32	45	41	Frontal_Mid_R
**7**	**7**	45	31	19	Frontal_Inf_Tri_R
**7**	**10**	45	20	33	Frontal_Inf_Oper_R
**8**	**6**	34	47	4	Frontal_Mid_R
**8**	**7**	51	51	3	Frontal_Inf_Tri_R
**9**	**7**	50	19	18	Frontal_Inf_Tri_R
**9**	**9**	68	3	25	Postcentral_R
**9**	**10**	64	17	37	Precentral_R
**10**	**9**	60	-14	43	Postcentral_R
**10**	**10**	50	-4	46	Precentral_R
**10**	**11**	55	-30	60	Parietal_Sup_R
**10**	**12**	42	-13	56	Precentral_R
**11**	**5**	23	28	46	Frontal_Sup_R
**11**	**10**	38	14	50	Frontal_Mid_R
**11**	**12**	31	2	62	Frontal_Sup_R
**12**	**11**	38	-39	56	Parietal_Inf_R
**12**	**12**	29	-26	56	Postcentral_R
**13**	**2**	-20	33	58	Frontal_Mid_L
**13**	**13**	-27	1	63	Precentral_L
**13**	**14**	-39	16	58	Frontal_Mid_L
**14**	**13**	-29	-23	76	Postcentral_L
**14**	**15**	-42	-38	69	Postcentral_L
**15**	**13**	-41	-9	67	Precentral_L
**15**	**14**	-34	-6	39	Precentral_L
**15**	**15**	-43	-26	48	Parietal_Inf_L
**15**	**16**	-59	-10	46	Postcentral_L
**16**	**1**	-39	27	20	Frontal_Inf_Tri_L
**16**	**14**	-44	18	29	Frontal_Inf_Tri_L
**16**	**16**	-43	8	24	Frontal_Inf_Oper_L

Supplemental Table 2. Detailed results of the Cambridge Neurospychological Test Automated Battery (CANTAB) cognitive test conducted before and after 24 hours of sleep deprivation

Descriptives

	N	Mean	Median	SD	SE
DMSCC_before	10	1.04000	1.02500	0.05164	0.0163
DMSCC_after	10	1.10500	1.10000	0.09560	0.0302
DMSL0SD_before	10	797.98116	624.17195	453.31492	143.3508
DMSL0SD_after	10	860.96673	773.69290	481.06734	152.1269
DMSL12SD_before	10	1007.45234	1081.56445	413.77131	130.8460
DMSL12SD_after	10	1215.67044	821.65655	1102.39109	348.6067
DMSL4SD_before	10	1205.95319	1278.37455	577.35857	182.5768
DMSL4SD_after	10	926.48694	933.90385	400.08461	126.5179
DMSLADSD_before	10	1074.66412	1107.65585	302.20743	95.5664
DMSLADSD_after	10	1169.65752	1050.48700	611.39796	193.3410
DMSLSD_before	10	1001.13844	1082.30140	280.89719	88.8275
DMSLSD_after	10	1229.61838	996.08700	702.92429	222.2842
DMSLSSD_before	10	710.94444	729.36430	369.39131	116.8118
DMSLSSD_after	10	710.50083	386.66095	1104.13917	349.1595
DMSMDL_before	10	2440.95000	2375.25000	526.99270	166.6497
DMSMDL_after	10	2422.40000	1991.00000	830.65399	262.6759
DMSMDL0_before	10	2491.60000	2386.50000	631.21804	199.6087
DMSMDL0_after	10	2187.20000	1967.50000	753.16411	238.1714
DMSMDL12_before	10	2718.35000	2394.00000	989.36736	312.8654
DMSMDL12_after	10	3367.00000	3170.00000	1386.95296	438.5930
DMSMDL4_before	10	2717.00000	2743.00000	698.12320	220.7659
DMSMDL4_after	10	2560.40000	2311.00000	803.63977	254.1332
DMSMDLAD_before	10	2562.85000	2423.25000	622.80927	196.9496
DMSMDLAD_after	10	2526.60000	2087.25000	749.85494	237.1250
DMSMDLS_before	10	2259.30000	2320.00000	490.80887	155.2074
DMSMDLS_after	10	2241.30000	2100.00000	891.35304	281.8706
DMSML_before	10	2703.16295	2686.50310	598.55367	189.2793
DMSML_after	10	2721.55231	2471.72445	892.30118	282.1704
DMSML0_before	10	2656.50000	2616.20000	625.30059	197.7374
DMSML0_after	10	2390.93333	2248.40000	796.83413	251.9811
DMSML12_before	10	2783.89833	2629.70000	791.31071	250.2344
DMSML12_after	10	3495.12500	3349.96665	1336.06649	422.5013
DMSML4_before	10	2973.53000	2860.75000	792.40446	250.5803
DMSML4_after	10	2666.51500	2292.20000	809.34922	255.9387
DMSMLAD_before	10	2795.90136	2835.26190	621.51436	196.5401
DMSMLAD_after	10	2809.16277	2568.84520	825.56764	261.0674
DMSMLS_before	10	2450.24000	2348.60000	602.09182	190.3982
DMSMLS_after	10	2402.20000	2060.80000	1335.10454	422.1971
DMSPC_before	10	96.00000	97.50000	5.16398	1.6330
DMSPC_after	10	91.50000	95.00000	8.51469	2.6926
DMSPC0_before	10	92.00000	100.00000	10.32796	3.2660
DMSPC0_after	10	92.00000	100.00000	13.98412	4.4222
DMSPC12_before	10	94.00000	100.00000	13.49897	4.2687
DMSPC12_after	10	80.00000	80.00000	21.08185	6.6667
DMSPC4_before	10	98.00000	100.00000	6.32456	2.0000
DMSPC4_after	10	96.00000	100.00000	8.43274	2.6667
DMSPCAD_before	10	94.60000	96.50000	6.86699	2.1715
DMSPCAD_after	10	89.30000	93.00000	10.85306	3.4320
DMSPCS_before	10	100.00000	100.00000	0.00000	0.0000
DMSPCS_after	10	98.00000	100.00000	6.32456	2.0000

Descriptives

	N	Mean	Median	SD	SE
DMSPEGC_before	10	0.04719	0.02780	0.06324	0.0200
DMSPEGC_after	10	0.08011	0.05560	0.08253	0.0261
DMSPEGE_before	3	0.00000	0.00000	0.00000	0.0000
DMSPEGE_after	3	0.19443	0.25000	0.17346	0.1001
DMSTC_before	10	19.20000	19.50000	1.03280	0.3266
DMSTC_after	10	18.30000	19.00000	1.70294	0.5385
DMSTC0_before	10	4.60000	5.00000	0.51640	0.1633
DMSTC0_after	10	4.60000	5.00000	0.69921	0.2211
DMSTC12_before	10	4.70000	5.00000	0.67495	0.2134
DMSTC12_after	10	4.00000	4.00000	1.05409	0.3333
DMSTC4_before	10	4.90000	5.00000	0.31623	0.1000
DMSTC4_after	10	4.80000	5.00000	0.42164	0.1333
DMSTCAD_before	10	14.20000	14.50000	1.03280	0.3266
DMSTCAD_after	10	13.40000	14.00000	1.64655	0.5207
DMSTCS_before	10	5.00000	5.00000	0.00000	0.0000
DMSTCS_after	10	4.90000	5.00000	0.31623	0.1000
DMSTE_before	10	0.80000	0.50000	1.03280	0.3266
DMSTE_after	10	1.70000	1.00000	1.70294	0.5385
DMSTEAD_before	10	0.80000	0.50000	1.03280	0.3266
DMSTEAD_after	10	1.60000	1.00000	1.64655	0.5207
DMSTEC_before	10	0.10000	0.00000	0.31623	0.1000
DMSTEC_after	10	0.50000	0.00000	0.70711	0.2236
DMSTECAD_before	10	0.10000	0.00000	0.31623	0.1000
DMSTECAD_after	10	0.50000	0.00000	0.70711	0.2236
DMSTED_before	10	0.00000	0.00000	0.00000	0.0000
DMSTED_after	10	0.00000	0.00000	0.00000	0.0000
DMSTEDAD_before	10	0.00000	0.00000	0.00000	0.0000
DMSTEDAD_after	10	0.00000	0.00000	0.00000	0.0000
DMSTEP_before	10	0.70000	0.50000	0.82327	0.2603
DMSTEP_after	10	1.20000	1.00000	1.54919	0.4899
DMSTEPAD_before	10	0.70000	0.50000	0.82327	0.2603
DMSTEPAD_after	10	1.10000	1.00000	1.52388	0.4819
MOTML_before	10	727.99000	689.00000	225.69214	71.3701
MOTML_after	10	717.61000	667.95000	236.84168	74.8959
MOTSDL_before	10	115.66900	110.36500	73.26198	23.1675
MOTSDL_after	10	102.47900	97.54500	46.32570	14.6495
MOTTC_before	10	10.00000	10.00000	0.00000	0.0000
MOTTC_after	10	10.00000	10.00000	0.00000	0.0000
MOTTE_before	10	0.00000	0.00000	0.00000	0.0000
MOTTE_after	10	0.00000	0.00000	0.00000	0.0000
PALFAMS_before	9	17.44444	19.00000	3.90868	1.3029
PALFAMS_after	9	16.88889	18.00000	3.40751	1.1358
PALMETS_before	9	0.44444	0.00000	0.72648	0.2422
PALMETS_after	9	0.77778	0.00000	1.09291	0.3643
PALNPR_before	9	8.22222	8.00000	1.56347	0.5212
PALNPR_after	9	8.00000	8.00000	0.00000	0.0000
PALTA_before	8	5.00000	5.00000	1.06904	0.3780
PALTA_after	8	5.75000	5.00000	1.66905	0.5901
PALTA2_before	10	1.00000	1.00000	0.00000	0.0000
PALTA2_after	10	1.00000	1.00000	0.00000	0.0000
PALTA4_before	10	1.10000	1.00000	0.31623	0.1000
PALTA4_after	10	1.10000	1.00000	0.31623	0.1000

Descriptives

	N	Mean	Median	SD	SE
PALTA6_before	10	1.30000	1.00000	0.94868	0.3000
PALTA6_after	10	1.40000	1.00000	0.51640	0.1633
PALTA8_before	10	1.70000	2.00000	0.94868	0.3000
PALTA8_after	10	2.10000	2.00000	1.19722	0.3786
PALTE_before	8	3.37500	1.00000	5.50162	1.9451
PALTE_after	8	6.62500	2.50000	9.21082	3.2565
PALTE2_before	10	0.00000	0.00000	0.00000	0.0000
PALTE2_after	10	0.00000	0.00000	0.00000	0.0000
PALTE4_before	10	0.10000	0.00000	0.31623	0.1000
PALTE4_after	10	0.10000	0.00000	0.31623	0.1000
PALTE6_before	10	1.60000	0.00000	5.05964	1.6000
PALTE6_after	10	0.70000	0.00000	1.05935	0.3350
PALTE8_before	10	1.50000	1.00000	1.71594	0.5426
PALTE8_after	10	4.80000	2.00000	7.50999	2.3749
PALTEA_before	8	6.87500	1.00000	15.14159	5.3534
PALTEA_after	8	6.62500	2.50000	9.21082	3.2565
PALTEA2_before	10	0.00000	0.00000	0.00000	0.0000
PALTEA2_after	10	0.00000	0.00000	0.00000	0.0000
PALTEA4_before	10	0.10000	0.00000	0.31623	0.1000
PALTEA4_after	10	0.10000	0.00000	0.31623	0.1000
PALTEA6_before	10	1.60000	0.00000	5.05964	1.6000
PALTEA6_after	10	0.70000	0.00000	1.05935	0.3350
PALTEA8_before	10	4.30000	1.50000	8.48594	2.6835
PALTEA8_after	10	4.80000	2.00000	7.50999	2.3749
RTIFESI_before	10	0.10000	0.00000	0.31623	0.1000
RTIFESI_after	10	0.30000	0.00000	0.48305	0.1528
RTIFESNR_before	10	0.00000	0.00000	0.00000	0.0000
RTIFESNR_after	10	0.00000	0.00000	0.00000	0.0000
RTIFESPR_before	10	0.20000	0.00000	0.42164	0.1333
RTIFESPR_after	10	0.10000	0.00000	0.31623	0.1000
RTIFMDMT_before	10	244.30000	240.00000	27.19395	8.5995
RTIFMDMT_after	10	286.65000	269.25000	62.27852	19.6942
RTIFMDRT_before	10	333.70000	326.25000	26.04718	8.2368
RTIFMDRT_after	10	367.35000	361.50000	49.49189	15.6507
RTIFMMT_before	10	247.27953	243.65170	26.36115	8.3361
RTIFMMT_after	10	291.86224	270.77760	60.68519	19.1903
RTIFMRT_before	10	341.19954	334.25000	22.61529	7.1516
RTIFMRT_after	10	378.46243	371.13335	55.28359	17.4822
RTIFMTSD_before	10	33.54375	31.36330	7.24862	2.2922
RTIFMTSD_after	10	48.39172	41.17155	22.60910	7.1496
RTIFRTSD_before	10	37.78215	38.50940	12.26708	3.8792
RTIFRTSD_after	10	58.21019	46.45715	32.19350	10.1805
RTIFTES_before	10	0.30000	0.00000	0.48305	0.1528
RTIFTES_after	10	0.50000	0.00000	0.70711	0.2236
RVPA_before	9	0.95699	0.96110	0.03731	0.0124
RVPA_after	9	0.92574	0.93680	0.04679	0.0156
RVPLSD_before	9	132.24538	143.24370	68.35897	22.7863
RVPLSD_after	9	146.53490	124.03920	82.88049	27.6268
RVPMDL_before	9	381.77778	383.00000	34.38669	11.4622
RVPMDL_after	9	394.33333	380.00000	32.42202	10.8073
RVPML_before	9	417.57419	426.39220	52.75695	17.5856
RVPML_after	9	439.24404	411.87800	62.61913	20.8730

Descriptives

	N	Mean	Median	SD	SE
RVPPFA_before	9	0.00232	0.00190	0.00229	7.65e-4
RVPPFA_after	9	0.00371	0.00370	0.00212	7.06e-4
RVPPH_before	9	0.83127	0.85190	0.14800	0.0493
RVPPH_after	9	0.70989	0.75930	0.18426	0.0614
RVPTFA_before	9	1.22222	1.00000	1.20185	0.4006
RVPTFA_after	9	1.88889	2.00000	1.05409	0.3514
RVPTH_before	9	44.88889	46.00000	7.99131	2.6638
RVPTH_after	9	38.33333	41.00000	9.94987	3.3166
RVPTM_before	9	9.11111	8.00000	7.99131	2.6638
RVPTM_after	9	15.66667	13.00000	9.94987	3.3166
SWMBE_before	8	0.62500	0.00000	1.40789	0.4978
SWMBE_after	8	1.25000	0.00000	2.81577	0.9955
SWMBE4_before	10	0.00000	0.00000	0.00000	0.0000
SWMBE4_after	10	0.00000	0.00000	0.00000	0.0000
SWMBE6_before	10	0.30000	0.00000	0.94868	0.3000
SWMBE6_after	10	1.00000	0.00000	2.53859	0.8028
SWMBE8_before	10	2.00000	0.00000	3.16228	1.0000
SWMBE8_after	10	1.70000	0.00000	4.71522	1.4911
SWMDE_before	8	0.00000	0.00000	0.00000	0.0000
SWMDE_after	8	0.00000	0.00000	0.00000	0.0000
SWMDE4_before	10	0.00000	0.00000	0.00000	0.0000
SWMDE4_after	10	0.00000	0.00000	0.00000	0.0000
SWMDE6_before	10	0.00000	0.00000	0.00000	0.0000
SWMDE6_after	10	0.00000	0.00000	0.00000	0.0000
SWMDE8_before	10	0.00000	0.00000	0.00000	0.0000
SWMDE8_after	10	0.50000	0.00000	1.58114	0.5000
SWMPR_before	10	5.20000	5.00000	0.42164	0.1333
SWMPR_after	10	5.00000	5.00000	0.00000	0.0000
SWMS_before	10	3.60000	2.50000	2.71621	0.8589
SWMS_after	10	3.70000	2.00000	2.75076	0.8699
SWMS6_before	10	1.80000	1.00000	1.31656	0.4163
SWMS6_after	10	1.90000	1.00000	1.66333	0.5260
SWMTE_before	8	0.87500	0.00000	2.10017	0.7425
SWMTE_after	8	1.25000	0.00000	2.81577	0.9955
SWMTE4_before	10	0.00000	0.00000	0.00000	0.0000
SWMTE4_after	10	0.00000	0.00000	0.00000	0.0000
SWMTE6_before	10	0.30000	0.00000	0.94868	0.3000
SWMTE6_after	10	1.00000	0.00000	2.53859	0.8028
SWMTE8_before	10	2.20000	0.00000	3.35989	1.0625
SWMTE8_after	10	1.90000	0.00000	5.34270	1.6895
SWMWE_before	8	0.25000	0.00000	0.70711	0.2500
SWMWE_after	8	0.00000	0.00000	0.00000	0.0000
SWMWE4_before	10	0.00000	0.00000	0.00000	0.0000
SWMWE4_after	10	0.00000	0.00000	0.00000	0.0000
SWMWE6_before	10	0.00000	0.00000	0.00000	0.0000
SWMWE6_after	10	0.00000	0.00000	0.00000	0.0000
SWMWE8_before	10	0.20000	0.00000	0.63246	0.2000
SWMWE8_after	10	0.70000	0.00000	2.21359	0.7000

Supplemental Table 3. Detailed results of the comparison of cognitive performance assessed before and after 24 hours of sleep deprivation via CANTAB cognitive test battery

Paired Samples T-Test

			statistic	p	Mean difference	SE difference
DMSCC_before	DMSCC_after	Wilcoxon W	5.00 [a]	0.076	-0.09459	0.0308
DMSL0SD_before	DMSL0SD_after	Wilcoxon W	23.00	0.695	-69.98700	216.9047
DMSL12SD_before	DMSL12SD_after	Wilcoxon W	28.00	1.000	11.55320	336.7195
DMSL4SD_before	DMSL4SD_after	Wilcoxon W	43.00	0.131	248.73010	165.6466
DMSLADSD_before	DMSLADSD_after	Wilcoxon W	30.00	0.846	29.44920	229.9429
DMSLSD_before	DMSLSD_after	Wilcoxon W	25.00	0.846	-39.64080	238.8677
DMSLSSD_before	DMSLSSD_after	Wilcoxon W	39.00	0.275	248.70630	346.9193
DMSMDL_before	DMSMDL_after	Wilcoxon W	31.00	0.770	78.00000	132.2127
DMSMDL0_before	DMSMDL0_after	Wilcoxon W	43.00	0.131	244.50000	161.0034
DMSMDL12_before	DMSMDL12_after	Wilcoxon W	16.00	0.275	-561.00000	442.3799
DMSMDL4_before	DMSMDL4_after	Wilcoxon W	35.00	0.492	183.75000	170.2885
DMSMDLAD_before	DMSMDLAD_after	Wilcoxon W	30.00	0.846	71.50000	145.4355
DMSMDLS_before	DMSMDLS_after	Wilcoxon W	38.00	0.322	94.00000	187.9914
DMSML_before	DMSML_after	Wilcoxon W	27.00	1.000	-49.25410	173.0338
DMSML0_before	DMSML0_after	Wilcoxon W	42.00	0.160	210.10000	187.4358
DMSML12_before	DMSML12_after	Wilcoxon W	12.00	0.131	-568.25000	382.4049
DMSML4_before	DMSML4_after	Wilcoxon W	42.00	0.160	321.70000	186.7359
DMSMLAD_before	DMSMLAD_after	Wilcoxon W	31.00	0.770	125.86190	198.0047
DMSMLS_before	DMSMLS_after	Wilcoxon W	42.00	0.160	285.30000	331.9875
DMSPC_before	DMSPC_after	Wilcoxon W	23.00 [b]	0.140	5.00001	2.5221
DMSPC0_before	DMSPC0_after	Wilcoxon W	7.50 [d]	1.000	-4.36e−5	5.9628
DMSPC12_before	DMSPC12_after	Wilcoxon W	15.00 [d]	0.053	29.99998	5.2068
DMSPC4_before	DMSPC4_after	Wilcoxon W	NaN [e]			
DMSPCAD_before	DMSPCAD_after	Wilcoxon W	23.00 [b]	0.143	6.99999	3.1342
DMSPCS_before	DMSPCS_after	Wilcoxon W	NaN [e]			
DMSPEGC_before	DMSPEGC_after	Wilcoxon W	10.00 [a]	0.288	-0.04026	0.0252
DMSPEGE_before	DMSPEGE_after	Wilcoxon W	NaN [f]			
DMSTC_before	DMSTC_after	Wilcoxon W	23.00 [b]	0.140	1.00005	0.5044
DMSTC0_before	DMSTC0_after	Wilcoxon W	7.50 [d]	1.000	-4.24e−5	0.2981
DMSTC12_before	DMSTC12_after	Wilcoxon W	15.00 [d]	0.053	1.49996	0.2603
DMSTC4_before	DMSTC4_after	Wilcoxon W	NaN [e]			
DMSTCAD_before	DMSTCAD_after	Wilcoxon W	23.00 [b]	0.143	1.00000	0.4667
DMSTCS_before	DMSTCS_after	Wilcoxon W	NaN [e]			
DMSTE_before	DMSTE_after	Wilcoxon W	5.00 [b]	0.140	-1.00005	0.5044
DMSTEAD_before	DMSTEAD_after	Wilcoxon W	5.00 [b]	0.143	-1.00000	0.4667
DMSTEC_before	DMSTEC_after	Wilcoxon W	NaN [e]			
DMSTECAD_before	DMSTECAD_after	Wilcoxon W	NaN [e]			
DMSTED_before	DMSTED_after	Wilcoxon W	NaN [e]			
DMSTEDAD_before	DMSTEDAD_after	Wilcoxon W	NaN [e]			
DMSTEP_before	DMSTEP_after	Wilcoxon W	7.50 [b]	0.301	-0.50003	0.4534
DMSTEPAD_before	DMSTEPAD_after	Wilcoxon W	9.00 [b]	0.430	-0.50002	0.4269
MOTML_before	MOTML_after	Wilcoxon W	32.00	0.695	12.50000	24.9260

[a] 2 pair(s) of values were tied

[b] 3 pair(s) of values were tied

[d] 5 pair(s) of values were tied

[e] All observations are tied

[f] f() values at end points not of opposite sign

[g] 4 pair(s) of values were tied

[h] 1 pair(s) of values were tied

[i] 6 pair(s) of values were tied

Paired Samples T-Test

			statistic	p	Mean difference	SE difference
MOTSDL_before	MOTSDL_after	Wilcoxon W	28.00	1.000	2.85500	24.2677
MOTTC_before	MOTTC_after	Wilcoxon W	NaN [e]			
MOTTE_before	MOTTE_after	Wilcoxon W	NaN [e]			
PALFAMS_before	PALFAMS_after	Wilcoxon W	18.50 [a]	0.495	0.86889	0.7093
PALMETS_before	PALMETS_after	Wilcoxon W	6.00 [b]	0.374	-0.50000	0.3333
PALNPR_before	PALNPR_after	Wilcoxon W	NaN [f]			
PALTA_before	PALTA_after	Wilcoxon W	2.00 [b]	0.170	-1.00010	0.4532
PALTA2_before	PALTA2_after	Wilcoxon W	NaN [e]			
PALTA4_before	PALTA4_after	Wilcoxon W	NaN [e]			
PALTA6_before	PALTA6_after	Wilcoxon W	NaN [f]			
PALTA8_before	PALTA8_after	Wilcoxon W	8.00 [g]	0.672	-0.99994	0.5812
PALTE_before	PALTE_after	Wilcoxon W	4.50 [h]	0.128	-3.75079	1.6771
PALTE2_before	PALTE2_after	Wilcoxon W	NaN [e]			
PALTE4_before	PALTE4_after	Wilcoxon W	NaN [e]			
PALTE6_before	PALTE6_after	Wilcoxon W	4.00 [i]	0.854	-0.99995	1.3618
PALTE8_before	PALTE8_after	Wilcoxon W	10.50 [a]	0.320	-2.78239	2.5519
PALTEA_before	PALTEA_after	Wilcoxon W	11.50 [h]	0.735	-0.99996	2.7042
PALTEA2_before	PALTEA2_after	Wilcoxon W	NaN [e]			
PALTEA4_before	PALTEA4_after	Wilcoxon W	NaN [e]			
PALTEA6_before	PALTEA6_after	Wilcoxon W	4.00 [i]	0.854	-0.99995	1.3618
PALTEA8_before	PALTEA8_after	Wilcoxon W	17.50 [a]	1.000	-2.60e−6	1.2134
RTIFESI_before	RTIFESI_after	Wilcoxon W	NaN [e]			
RTIFESNR_before	RTIFESNR_after	Wilcoxon W	NaN [e]			
RTIFESPR_before	RTIFESPR_after	Wilcoxon W	NaN [e]			
RTIFMDMT_before	RTIFMDMT_after	Wilcoxon W	7.00	0.037	-35.50000	16.1297
RTIFMDRT_before	RTIFMDRT_after	Wilcoxon W	8.50	0.059	-30.24996	14.4442
RTIFMMT_before	RTIFMMT_after	Wilcoxon W	4.00	0.014	-35.48535	15.9774
RTIFMRT_before	RTIFMRT_after	Wilcoxon W	6.00	0.027	-32.58850	14.8677
RTIFMTSD_before	RTIFMTSD_after	Wilcoxon W	10.00	0.084	-12.77440	6.7662
RTIFRTSD_before	RTIFRTSD_after	Wilcoxon W	4.00	0.014	-15.34170	8.4950
RTIFTES_before	RTIFTES_after	Wilcoxon W	NaN [f]			
RVPA_before	RVPA_after	Wilcoxon W	44.00	0.008	0.02575	0.0126
RVPLSD_before	RVPLSD_after	Wilcoxon W	21.00	0.910	-2.79880	25.3841
RVPMDL_before	RVPMDL_after	Wilcoxon W	12.00	0.250	-13.25000	9.3190
RVPML_before	RVPML_after	Wilcoxon W	14.00	0.359	-16.70455	19.0522
RVPPFA_before	RVPPFA_after	Wilcoxon W	4.00 [a]	0.108	-0.00189	9.63e-4
RVPPH_before	RVPPH_after	Wilcoxon W	44.00	0.013	0.10178	0.0493
RVPTFA_before	RVPTFA_after	Wilcoxon W	7.00 [a]	0.240	-0.99995	0.5000
RVPTH_before	RVPTH_after	Wilcoxon W	44.00	0.013	5.49994	2.6620
RVPTM_before	RVPTM_after	Wilcoxon W	1.00	0.013	-5.49996	2.6620
SWMBE_before	SWMBE_after	Wilcoxon W	4.00 [g]	0.855	-1.12941	1.2092
SWMBE4_before	SWMBE4_after	Wilcoxon W	NaN [e]			
SWMBE6_before	SWMBE6_after	Wilcoxon W	NaN [f]			

[a] 2 pair(s) of values were tied
[b] 3 pair(s) of values were tied
[d] 5 pair(s) of values were tied
[e] All observations are tied
[f] f() values at end points not of opposite sign
[g] 4 pair(s) of values were tied
[h] 1 pair(s) of values were tied
[i] 6 pair(s) of values were tied

Paired Samples T-Test

			statistic	p	Mean difference	SE difference
SWMBE8_before	SWMBE8_after	Wilcoxon W	8.50 [d]	0.892	0.99996	1.2828
SWMDE_before	SWMDE_after	Wilcoxon W	NaN [e]			
SWMDE4_before	SWMDE4_after	Wilcoxon W	NaN [e]			
SWMDE6_before	SWMDE6_after	Wilcoxon W	NaN [e]			
SWMDE8_before	SWMDE8_after	Wilcoxon W	NaN [e]			
SWMPR_before	SWMPR_after	Wilcoxon W	NaN [e]			
SWMS_before	SWMS_after	Wilcoxon W	10.00 [d]	0.583	0.99998	0.6904
SWMS6_before	SWMS6_after	Wilcoxon W	6.00 [i]	0.854	0.99995	0.5859
SWMTE_before	SWMTE_after	Wilcoxon W	4.00 [g]	0.855	-0.75834	1.3620
SWMTE4_before	SWMTE4_after	Wilcoxon W	NaN [e]			
SWMTE6_before	SWMTE6_after	Wilcoxon W	NaN [f]			
SWMTE8_before	SWMTE8_after	Wilcoxon W	8.00 [d]	1.000	1.00002	1.5059
SWMWE_before	SWMWE_after	Wilcoxon W	NaN [e]			
SWMWE4_before	SWMWE4_after	Wilcoxon W	NaN [e]			
SWMWE6_before	SWMWE6_after	Wilcoxon W	NaN [e]			
SWMWE8_before	SWMWE8_after	Wilcoxon W	NaN [f]			

[a] 2 pair(s) of values were tied

[b] 3 pair(s) of values were tied

[d] 5 pair(s) of values were tied

[e] All observations are tied

[f] f() values at end points not of opposite sign

[g] 4 pair(s) of values were tied

[h] 1 pair(s) of values were tied

[i] 6 pair(s) of values were tied

Supplemental Table 4. Abbreviations and measure descriptions of parameters assessed by CANTAB cognitive test battery

Task	Measure Abbreaviation	Measure Description
DMS	DMSCC	DMS Mean Choices to Correct: The mean number of choices that the subject made on each trial, including the correct choice. Calculated across all trials where the subject eventually made the correct choice (simultaneous and all delays).
DMS	DMSL0SD	DMS Correct Latency Standard Deviation (SD) (0 second delay): The standard deviation of response latencies for trials containing a zero second delay between the presentation of target and response stimuli, where subjects selected the correct box on their first attempt. Calculated across all assessed trials containing a zero second delay.
DMS	DMSL12SD	DMS Correct Latency Standard Deviation (SD) (12 second delay): The standard deviation of response latencies for trials containing a twelve second delay between the presentation of target and response stimuli, where subjects selected the correct box on their first attempt. Calculated across all assessed trials containing a twelve second delay.
DMS	DMSL4SD	DMS Correct Latency Standard Deviation (SD) (4 second delay): The standard deviation of response latencies for trials containing a four second delay between the presentation of target and response stimuli, where subjects selected the correct box on their first attempt. Calculated across all assessed trials containing a four second delay.
DMS	DMSLADSD	DMS Correct Latency Standard Deviation (SD) (all delays): The standard deviation of response latencies for trials containing a delay between the presentation of target stimulus and response stimuli, where subjects selected the correct box on their first attempt. Calculated across all assessed trials containing a delay.
DMS	DMSLSD	DMS Correct Latency Standard Deviation (SD): The standard deviation of response latencies for trials where subjects selected the correct box on their first attempt. Calculated across all correct assessed trials (simultaneous and all delays).
DMS	DMSLSSD	DMS Correct Latency Standard Deviation (SD) (simultaneous): The standard deviation of response latencies for trials containing a simultaneous presentation of target and response stimuli, where subjects selected the correct box on their first attempt. Calculated across all assessed trials containing simultaneous presentations.
DMS	DMSMDL	DMS Median Correct Latency: The median latency between the presentation of the response stimuli options and the subject selecting the correct box on their first attempt. Calculated across all correct assessed trials (simultaneous and all delays).
DMS	DMSMDL0	DMS Median Correct Latency (0 seconds delay): The median latency between the presentation of the response stimuli options and the subject selecting the correct box on their first attempt for trials containing a zero second delay. Calculated across all assessed trials containing a zero second delay.

DMS	DMSMDL12	DMS Median Correct Latency (12 seconds delay): The median latency between the presentation of the response stimuli options and the subject selecting the correct box on their first attempt for trials containing a twelve second delay. Calculated across all assessed trials containing a twelve second delay.
DMS	DMSMDL4	DMS Median Correct Latency (4 seconds delay): The median latency between the presentation of the response stimuli options and the subject selecting the correct box on their first attempt for trials containing a four second delay. Calculated across all assessed trials containing a four second delay.
DMS	DMSMDLAD	DMS Median Correct Latency (all delays): The median latency between the presentation of the response stimuli options and the subject selecting the correct box on their first attempt for trials containing a delay between target and response stimuli presentation. Calculated across all assessed trials containing a delay.
DMS	DMSMDLS	DMS Median Correct Latency (simultaneous): The median latency between the presentation of the response stimuli options and the subject selecting the correct box on their first attempt for trials containing a simultaneous presentation of target and response stimuli. Calculated across all assessed trials containing simultaneous presentation.
DMS	DMSML	DMS Mean Correct Latency: The mean latency between the presentation of the response stimuli options and the subject selecting the correct box on their first attempt. Calculated across all correct assessed trials (simultaneous and all delays).
DMS	DMSML0	DMS Mean Correct Latency (0 seconds delay): The mean latency between the presentation of the response stimuli options and the subject selecting the correct box on their first attempt for trials containing a zero second delay. Calculated across all assessed trials containing a zero second delay.
DMS	DMSML12	DMS Mean Correct Latency (12 seconds delay): The mean latency between the presentation of the response stimuli options and the subject selecting the correct box on their first attempt for trials containing a twelve second delay. Calculated across all assessed trials containing a twelve second delay.
DMS	DMSML4	DMS Mean Correct Latency (4 seconds delay): The mean latency between the presentation of the response stimuli options and the subject selecting the correct box on their first attempt for trials containing a four second delay. Calculated across all assessed trials containing a four second delay.
DMS	DMSMLAD	DMS Mean Correct Latency (all delays): The mean latency between the presentation of the response stimuli options and the subject selecting the correct box on their first attempt for trials containing a delay between target and response stimuli presentation. Calculated across all assessed trials containing a delay.
DMS	DMSMLS	DMS Mean Correct Latency (simultaneous): The mean latency between the presentation of the response stimuli options and the subject selecting the correct box on their first attempt for trials containing a simultaneous presentation of target and response stimuli. Calculated across all assessed trials containing simultaneous presentation.
DMS	DMSPC	DMS Percent Correct: The percentage of assessment trials during which the subject chose the correct box on their first box choice. Calculated across all assessed trials (simultaneous presentation and all delays).

DMS	DMSPC0	KEY: DMS Percent Correct (0 seconds delay): The percentage of assessment trials containing a zero second delay during which the subject chose the correct box on their first box choice. Calculated across all assessed trials containing a zero second delay.
DMS	DMSPC12	KEY: DMS Percent Correct (12 second delay): The percentage of assessment trials containing a twelve second delay during which the subject chose the correct box on their first box choice. Calculated across all assessed trials containing a twelve second delay.
DMS	DMSPC4	KEY: DMS Percent Correct (4 second delay): The percentage of assessment trials containing a four second delay during which the subject chose the correct box on their first box choice. Calculated across all assessed trials containing a four second delay.
DMS	DMSPCAD	KEY: DMS Percent Correct (all delays): The percentage of assessment trials containing a delay during which the subject chose the correct box on their first box choice. Calculated across all assessed trials containing a delay.
DMS	DMSPCS	KEY: DMS Percent Correct (simultaneous): The percentage of assessment trials where the target and response stimuli were presented simultaneously during which the subject chose the correct box on their first box choice. Calculated across all assessed trials containing the simultaneous presentation of stimuli.
DMS	DMSPEGC	DMS Probability of Error Given Correct: This measure reports the probability of an error being made when the previous trial was responsed to correctly by the subject. Calculated across all assessed trials (simultaneous and all delays).
DMS	DMSPEGE	KEY: DMS Probability of Error Given Error: This measure reports the probability of an error occurring when the previous trial was responded to incorrectly. Calculated across all assessed trials (simultaneous and all delays).
DMS	DMSTC	DMS Total Correct: The total number of times a subject chose the correct answer on their first box choice. Calculated across all assessed trials (simultaneous presentation and all delays).
DMS	DMSTC0	DMS Total Correct (0 second delay): The total number of times a subject chose the correct answer on their first box choice for trials where the response stimuli appeared on screen after a 0 second delay after the target stimulus was shown. Calculated across all assessed trials which contained a delay of zero seconds.
DMS	DMSTC12	DMS Total Correct (12 second delay): The total number of times a subject chose the correct answer on their first box choice for trials where the response stimuli appeared on screen after a 12 second delay after the target stimulus was shown. Calculated across all assessed trials which contained a delay of twelve seconds.
DMS	DMSTC4	DMS Total Correct (4 second delay): The total number of times a subject chose the correct answer on their first box choice for trials where the response stimuli appeared on screen after a 4 second delay after the target stimulus was shown. Calculated across all assessed trials which contained a delay of four seconds.
DMS	DMSTCAD	DMS Total Correct (all delays): The total number of times a subject chose the correct answer on their first box choice for all trials where the response stimuli were presented after a delay. Calculated across all assessed trials containing a delay.

DMS	DMSTCS	DMS Total Correct (simultaneous): The total number of times a subject chose the correct answer on their first box choice for trials where the target stimulus and response stimuli appeared on screen simultaneously. Calculated across all assessed trials that included a simultaneous presentation (no delay) of target and response stimuli.
DMS	DMSTE	DMS Total Errors: The total number of times a subject failed to choose the correct box on their first selection, thus making an error. Calculated across all assessed trials (simultaneous and all delays) regardless of which incorrect box (out of the 3 possible incorrect boxes) was chosen.
DMS	DMSTEAD	DMS Total Errors (all delays): The total number of times a subject failed to choose the correct box on their first selection for any trial containing a delay between the presentation of the target stimulus and response stimuli. Calculated across all assessed trials containing a delay component.
DMS	DMSTEC	DMS Error (incorrect colour): The number of times that the subject failed to select the correct box on their first selection, and instead chose the distractor stimulus that contained the same colour elements, but different physical attributes. Calculated across all assessed trials (simultaneous and all delays).
DMS	DMSTECAD	DMS Error (all delays, incorrect colour): The number of times that the subject failed to select the correct box on their first selection, and instead chose the distractor stimulus that contained the same colour elements, but different physical attributes. Calculated across all assessed trials which contained a delay component.
DMS	DMSTED	DMS Error (distractor): The number of times that the subject failed to select the correct box on their first selection, and instead chose the distractor stimulus that contained no common elements to the original target stimulus. Calculated across all assessed trials (simultaneous and all delays).
DMS	DMSTEDAD	DMS Error (all delays, distractor): The number of times that the subject failed to select the correct box on their first selection, and instead chose the distractor stimulus that contained no common elements to the original target stimulus. Calculated across all assessed trials which contained a delay component.
DMS	DMSTEP	DMS Error (incorrect pattern): The number of times that the subject failed to select the correct box on their first selection, and instead chose the distractor stimulus that contained the same pattern/ physical attributes, but different colours. Calculated across all assessed trials (simultaneous and all delays).
DMS	DMSTEPAD	DMS Error (all delays, incorrect pattern): The number of times that the subject failed to select the correct box on their first selection, and instead chose the distractor stimulus that contained the same pattern/ physical attributes, but different colour elements. Calculated across all assessed trials which contained a delay component.
MOT	MOTML	The mean latency from the display of a stimulus to a correct response to that stimulus during assessment trials.
MOT	MOTSDL	This is the standard deviation of the latency, calculated from the display of a stimulus to a correct response to that stimulus during assessment trials.
MOT	MOTTC	The total number of assessment trials on which the subject made a correct response.
MOT	MOTTE	The total number of assessment trials on which the subject failed to make a correct response.

PAL	PALFAMS28	KEY: PAL First Attempt Memory Score: The number of times a subject chose the correct box on their first attempt when recalling the pattern locations. Calculated across assessed trials, omitting 12 box level to provide a direct comparison to Recommended Standard..
PAL	PALMETS28	PAL Mean Errors to Success: The mean number of attempts made by a subject needed for them to successfully complete the stage. Does not include 12 box level to provide a direct comparison to Recommended Standard.
PAL	PALNPR28	PAL Number of Patterns Reached: The number of patterns presented to the subject on the last problem they reached.
PAL	PALTA12	PAL Total Attempts 12 Patterns: The total number of attempts made (but not necessarily completed) by the subject during assessment problems containing a total of 12 shapes to recall.
PAL	PALTA2	PAL Total Attempts 2 Patterns: The total number of attempts made (but not necessarily completed) by the subject during assessment problems containing a total of 2 shapes to recall.
PAL	PALTA28	PAL Total Attempts: The total number of attempts made (but not necessarily completed) by the subject during assessment problems. Does not include 12 box level to provide a direct comparison to Recommended Standard.
PAL	PALTA4	PAL Total Attempts 4 patterns: The total number of attempts made (but not necessarily completed) by the subject during assessment problems containing a total of 4 shapes to recall.
PAL	PALTA6	PAL Total Attempts 6 Patterns: The total number of attempts made (but not necessarily completed) by the subject during assessment problems containing a total of 6 shapes to recall.
PAL	PALTA8	PAL Total Attempts 8 Patterns: The total number of attempts made (but not necessarily completed) by the subject during assessment problems containing a total of 8 shapes to recall.
PAL	PALTE12	PAL Total Errors 12 Patterns: The total number of times a subject selected an incorrect box when attempting to recall a pattern location on trials containing a total of 12 patterns. Calculated across all 12-pattern assessed trials.
PAL	PALTE2	PAL Total Errors 2 Patterns: The total number of times a subject selected an incorrect box when attempting to recall a pattern location on trials containing a total of 2 patterns. Calculated across all 2-pattern assessed trials.
PAL	PALTE28	PAL Total Errors: The total number of times a subject selected an incorrect box when attempting to recall a pattern location. Calculated across all assessed trials. Does not include 12 box level to provide a direct comparison to Recommended Standard.
PAL	PALTE4	PAL Total Errors 4 Patterns: The total number of times a subject selected an incorrect box when attempting to recall a pattern location on trials containing a total of 4 patterns. Calculated across all 4-pattern assessed trials.
PAL	PALTE6	PAL Total Errors 6 Patterns: The total number of times a subject selected an incorrect box when attempting to recall a pattern location on trials containing a total of 6 patterns. Calculated across all 6-pattern assessed trials.
PAL	PALTE8	PAL Total Errors 8 Patterns: The total number of times a subject selected an incorrect box when attempting to recall a pattern location on trials containing a total of 8 patterns. Calculated across all 8-pattern assessed trials.

PAL	PALTEA12	PAL Total Errors 12 Shapes (Adjusted): The number of times the subject chose the incorrect box for a stimulus on assessment problems, where the number of shapes was equal to 12 (PALTE12), plus an adjustment for the estimated number of errors they would have made on any other 12 pattern problems, attempts and recalls they did not reach.
PAL	PALTEA2	PAL Total Errors 2 Shapes (Adjusted): The number of times the subject chose the incorrect box for a stimulus on assessment problems, where the number of shapes required to remember was equal to 2 (PALTE2), plus an adjustment for the estimated number of errors they would have made on any other 2 pattern problems, attempts and recalls they did not reach.
PAL	PALTEA28	KEY: PAL Total Errors (Adjusted): The number of times the subject chose the incorrect box for a stimulus on assessment problems (PALTE), plus an adjustment for the estimated number of errors they would have made on any problems, attempts and recalls they did not reach. This measure allows you to compare performance on errors made across all subjects regardless of those who terminated early versus those completing the final stage of the task. In this task variant PALTEA does not include 12 box level to provide a direct comparison to Recommended Standard.
PAL	PALTEA4	PAL Total Errors 4 Shapes (Adjusted): The number of times the subject chose the incorrect box for a stimulus on assessment problems, where the number of shapes was equal to 4 (PALTE4), plus an adjustment for the estimated number of errors they would have made on any other 4 pattern problems, attempts and recalls they did not reach.
PAL	PALTEA6	PAL Total Errors 6 Shapes (Adjusted): The number of times the subject chose the incorrect box for a stimulus on assessment problems, where the number of shapes was equal to 6 (PALTE6), plus an adjustment for the estimated number of errors they would have made on any other 6 pattern problems, attempts and recalls they did not reach.
PAL	PALTEA8	PAL Total Errors 8 Shapes (Adjusted): The number of times the subject chose the incorrect box for a stimulus on assessment problems, where the number of shapes was equal to 8 (PALTE8), plus an adjustment for the estimated number of errors they would have made on any other 8 pattern problems, attempts and recalls they did not reach.
RTI	RTIFESI	RTI Five-Choice Error Score (inaccurate): The total number of trials where the subject made an inaccurate response. Calculated across all assessment trials in which the stimulus could appear in any one of five locations.
RTI	RTIFESNR	RTI Five-Choice Error Score (no response): The total number of trials where the subject made no response after the presentation of the target stimulus. Calculated across all assessment trials in which the stimulus could appear in any one of five locations.
RTI	RTIFESPR	RTI Five-Choice Error Score (premature): The total number of trials where the subject made a response before the presentation of the target stimulus. Calculated across all assessment trials in which the stimulus could appear in any one of five locations.

RTI	RTIFMDMT	KEY: RTI Median Five-Choice Movement Time: The median time taken for a subject to release the response button and select the target stimulus after it flashed yellow on screen. Calculated across correct, assessed trials in which the stimulus could appear in any one of five locations. Measured in milliseconds.
RTI	RTIFMDRT	KEY: RTI Median Five-Choice Reaction Time: The median duration it took for a subject to release the response button after the presentation of a target stimulus. Calculated across correct, assessed trials in which the stimulus could appear in any one of five locations. Measured in milliseconds.
RTI	RTIFMMT	RTI Mean Five-Choice Movement Time: The mean time taken for a subject to release the response button and select the target stimulus after it flashed yellow on screen. Calculated across correct, assessed trials in which the stimulus could appear in any one of five locations. Measured in milliseconds.
RTI	RTIFMRT	RTI Mean Five-Choice Reaction Time: The mean duration it took for a subject to release the response button after the presentation of a target stimulus. Calculated across correct, assessed trials in which the stimulus could appear in any one of five locations. Measured in milliseconds.
RTI	RTIFMTSD	RTI Standard Deviation (SD) Five-Choice Movement Time: The standard deviation calculated for the duration it took for a subject to release the response button and select the target stimulus after it flashed yellow on screen.Calculated across correct, assessed trials in which the stimulus could appear in any one of five locations. Measured in milliseconds.
RTI	RTIFRTSD	RTI Standard Deviation (SD) Five-Choice Reaction Time: The standard deviation calculated for the duration it took for a subject to release the response button after the presentation of a target stimulus. Calculated across correct, assessed trials in which the stimulus could appear in any one of five locations. Measured in milliseconds.
RTI	RTIFTES	RTI Total Error Score (Five-Choice): The total number of trials where the subject made any form of response error. This measure is calculated through summing the inaccurate, incorrect location, omission and premature errors, alongside two other possible errors not individually output. These two additional errors are the use of multiple fingers and dragging a finger outside of a response box. Calculated across all assessment trials in which the stimulus could appear in any one of five locations. Please note that this outcome measure combines multiple, separate cognitive functions (i.e. an omission error is not psychologically the same as a premature error) and is therefore not a recommended measure for standard practice.
RVP	RVPA	KEY: RVP A?: A? (A prime) is the signal detection measure of a subject's sensitivity to the target sequence (string of three numbers), regardless of response tendency (the expected range is 0.00 to 1.00; bad to good). In essence, this metric is a measure of how good the subject is at detecting target sequences.
RVP	RVPLSD	RVP Response Latency (SD): The standard deviation of response latency on trials where the subject responded correctly. Calculated across all assessed trials.
RVP	RVPMDL	KEY: RVP Median Response Latency: The median response latency on trials where the subject responded correctly. Calculated across all assessed trials.

RVP	RVPML	RVP Mean Response Latency: The mean response latency on trials where the subject responded correctly. Calculated across all assessed trials.
RVP	RVPPFA	KEY: RVP Probability of False Alarm: The number of sequence presentations that were false alarms divided by the number of sequence presentations that were false alarms plus the number of sequence presentations that were correct rejections: (False Alarms ÷ (False Alarms + Correct Rejections))
RVP	RVPPH	RVP Probability of Hit: The number of target sequences during assessment blocks that were correctly responded to within the time allowed, divided by the number of target sequences during assessment blocks (Correct hits ÷ total number of sequences)
RVP	RVPTFA	RVP Total False Alarms: The total number of stimulus presentations during assessment blocks that were false alarms.
RVP	RVPTH	RVP Total Hits: The total number of target sequences that were correctly responded to (Correct Hits) within the allowed time during assessment sequence blocks.
RVP	RVPTM	RVP Total Misses: The total number of target sequences that were not responded to within the allowed time during assessment sequence blocks.
SWM	SWMBE12	KEY: SWM Between errors 12 boxes: The number of times the subject revisits a box in which a token has previously been found. Calculated across all trials with 12 tokens only.
SWM	SWMBE4	KEY: SWM Between errors 4 boxes: The number of times a subject revisits a box in which a token has previously been found. Calculated across all trials with 4 tokens only.
SWM	SWMBE468	KEY: SWM Between Errors: The number of times the subject incorrectly revisits a box in which a token has previously been found. Calculated across all assessed four, six and eight token trials.
SWM	SWMBE6	KEY: SWM Between errors 6 boxes: The number of times the subject revisits a box in which a token has previously been found. Calculated across all trials with 6 tokens only.
SWM	SWMBE8	KEY: SWM Between errors 8 boxes: The number of times the subject revisits a box in which a token has previously been found. Calculated across all trials with 8 tokens only.
SWM	SWMDE12	SWM Double errors 12 boxes: The number of times a subject commits an error that is both a within error and a between error. Calculated across all trials with 12 tokens only.
SWM	SWMDE4	SWM Double errors 4 boxes: The number of times a subject commits an error that is both a within error and a between error. Calculated across all trials with 4 tokens only.
SWM	SWMDE468	SWM Double Errors: The number of times a subject commits an error that is both a within error and a between error. Calculated across all assessed four, six and eight token trials.
SWM	SWMDE6	SWM Double errors 6 boxes: The number of times a subject commits an error that is both a within error and a between error. Calculated across all trials with 6 tokens only.
SWM	SWMDE8	SWM Double errors 8 boxes: The number of times a subject commits an error that is both a within error and a between error. Calculated across all trials with 8 tokens only.

SWM	SWMPR	SWM Problem Reached: This measure reports the problem number that the subject reached, but did not necessarily complete.
SWM	SWMS	KEY: SWM Strategy (6-8 boxes): The number of times a subject begins a new search pattern from the same box they started with previously. If they always begin a search from the same starting point we infer that the subject is employing a planned strategy for finding the tokens. Therefore a low score indicates high strategy use (1 = they always begin the search from the same box), a high score indicates that they are beginning their searches from many different boxes. Calculated across assessed trials with 6 tokens or 8 tokens.
SWM	SWMS6	SWM Strategy (6 box only): This measure computes the strategy score for the 6 box stage of the task only. The strategy score is calculated based on the number of times a subject begins a new search pattern from the same box they started with previously. If they always begin a search from the same starting point we infer that the subject is employing a planned strategy for finding the tokens. Therefore a low score indicates high strategy use (1 = they always begin the search from the same box), a high score indicates that they are beginning their searches from many different boxes.
SWM	SWMSX	SWM Strategy (6-12 boxes): The number of times a subject begins a new search pattern from the same box they started with previously. If they always begin a search from the same starting point we infer that the subject is employing a planned strategy for finding the tokens. Therefore a low score indicates high strategy use (1 = they always begin the search from the same box), a high score indicates that they are beginning their searches from many different boxes. Calculated across assessed trials with 6 tokens or more.
SWM	SWMTE12	SWM Total errors 12 boxes: The number of times a box is selected that is certain not to contain a token and therefore should not have been visited by the subject, i.e. between errors + within errors - double errors. Calculated across all trials with 12 tokens only.
SWM	SWMTE4	SWM Total errors 4 boxes: The number of times a box is selected that is certain not to contain a token and therefore should not have been visited by the subject, i.e. between errors + within errors - double errors. Calculated across all trials with 4 tokens only.
SWM	SWMTE468	SWM Total Errors: The total number of times a box is selected that is certain not to contain a token and therefore should not have been visited by the subject, i.e. between errors + within errors - double errors. Calculated across all assessed four, six and eight token trials.
SWM	SWMTE6	SWM Total errors 6 boxes: The number of times a box is selected that is certain not to contain a token and therefore should not have been visited by the subject, i.e. between errors + within errors - double errors. Calculated across all trials with 6 tokens only.
SWM	SWMTE8	SWM Total errors 8 boxes: The number of times a box is selected that is certain not to contain a token and therefore should not have been visited by the subject, i.e. between errors + within errors - double errors. Calculated across all trials with 8 tokens only.

SWM	SWMWE12	SWM Within errors 12 boxes: The number of times a subject revisits a box already found to be empty during the same search. Calculated across all trials with 12 tokens only.
SWM	SWMWE4	SWM Within errors 4 boxes: The number of times a subject revisits a box already found to be empty during the same search. Calculated across all trials with 4 tokens only.
SWM	SWMWE468	SWM Within Errors: The number of times a subject revisits a box already shown to be empty during the same search. Calculated across all assessed four, six and eight token trials.
SWM	SWMWE6	SWM Within errors 6 boxes: The number of times a subject revisits a box already found to be empty during the same search. Calculated across all trials with 6 tokens only.
SWM	SWMWE8	SWM Within errors 8 boxes: The number of times a subject revisits a box already found to be empty during the same search. Calculated across all trials with 8 tokens only.

Supplemental Table 5. Numerical results of the general linear model (GLM)-based statistical approach to detect neurovascular coupling (NVC) on the group level. Rows highlighted with bold font indicate significant NVC responses.

source	detector	type	cond	beta	se	tstat	dfe	p	q	power
1	1	hbo	before	3.97292975	2.516794204	1.578567586	34	0.12369515	0.26168646	0.32692129
**1**	**2**	**hbo**	**before**	**10.57748046**	**2.856508445**	**3.702940376**	**34**	**0.00075187**	**0.01389614**	**0.87189107**
1	14	hbo	before	-0.397110413	2.607231491	-0.152311145	34	0.8798418	0.63759706	0.68826999
2	1	hbo	before	0.545057197	2.468234925	0.220828735	34	0.82654657	0.62307219	0.62541007
2	3	hbo	before	4.541410774	3.255970729	1.394794718	34	0.17212414	0.30927101	0.33929215
3	1	hbo	before	8.416798511	4.59700722	1.830930018	34	0.07588691	0.21638609	0.31359529
**3**	**2**	**hbo**	**before**	**8.029312307**	**2.563345157**	**3.132357063**	**34**	**0.00355695**	**0.03134536**	**0.71883085**
**3**	**3**	**hbo**	**before**	**5.934688767**	**1.682613052**	**3.52706688**	**34**	**0.00122605**	**0.01710437**	**0.83282486**
**3**	**4**	**hbo**	**before**	**15.81091745**	**3.213702013**	**4.919845518**	**34**	**2.1815E-05**	**0.00214569**	**0.98808928**
4	2	hbo	before	5.864665717	2.358464835	2.486645393	34	0.01796642	0.0841635	0.47616886
**4**	**4**	**hbo**	**before**	**17.2390509**	**4.274802235**	**4.032713083**	**34**	**0.00029481**	**0.0083465**	**0.92644421**
**4**	**5**	**hbo**	**before**	**12.37773301**	**2.587542261**	**4.783586801**	**34**	**3.2717E-05**	**0.00214569**	**0.98379893**
**5**	**3**	**hbo**	**before**	**8.719738843**	**2.382364144**	**3.660120081**	**34**	**0.00084755**	**0.01389614**	**0.86304944**
**5**	**4**	**hbo**	**before**	**8.701950813**	**2.3723164**	**3.668124038**	**34**	**0.00082881**	**0.01389614**	**0.86473451**
5	6	hbo	before	2.76835555	2.458839076	1.12587911	34	0.26810135	0.36630939	0.36341397
**6**	**4**	**hbo**	**before**	**8.039166574**	**2.795670177**	**2.875577612**	**34**	**0.00691286**	**0.04772265**	**0.62773444**
6	5	hbo	before	1.032242809	2.773322732	0.372204359	34	0.71205067	0.59621561	0.53335885
6	6	hbo	before	-3.282869178	2.59855342	-1.263344887	34	0.21505987	0.33682408	0.35001382
**6**	**7**	**hbo**	**before**	**-12.46252083**	**3.708662946**	**-3.36038109**	**34**	**0.00193373**	**0.02113659**	**0.78902654**
7	5	hbo	before	3.117658729	3.714117226	0.839407735	34	0.40710415	0.4381224	0.40188662
7	7	hbo	before	2.938598913	2.057734473	1.428074881	34	0.16239223	0.3000041	0.33684667
7	10	hbo	before	3.539558997	2.938733047	1.204450673	34	0.23673315	0.34246589	0.3554473
8	6	hbo	before	-4.547545153	3.010581441	-1.510520556	34	0.14014954	0.27437034	0.33119384
8	7	hbo	before	-2.483428109	2.113188963	-1.175203994	34	0.24807771	0.3498848	0.35831131
9	7	hbo	before	5.962853277	4.702744659	1.267951741	34	0.2134296	0.33682408	0.34960653
9	9	hbo	before	3.165634194	3.413107029	0.927493386	34	0.36020815	0.415002	0.38814058
9	10	hbo	before	2.572152819	3.18801297	0.806820061	34	0.42537827	0.44995999	0.40752211
10	9	hbo	before	6.455281438	4.127865478	1.56383038	34	0.1271185	0.26466011	0.32781863
**10**	**10**	**hbo**	**before**	**17.11883228**	**4.884565778**	**3.504678421**	**34**	**0.00130403**	**0.01710437**	**0.82732554**
10	11	hbo	before	8.625897837	4.017301321	2.147187166	34	0.03899565	0.14613977	0.34601648
10	12	hbo	before	4.347157481	3.383739447	1.284719923	34	0.20757433	0.33682408	0.34814471
11	5	hbo	before	5.662279366	4.515962953	1.25383654	34	0.21845429	0.33682408	0.35086233

11	10	hbo	before	2.679063416	2.891437484	0.926550697	34	0.36069055	0.415002	0.38827728
11	12	hbo	before	3.678865876	4.093141486	0.89878786	34	0.37508707	0.42492935	0.3923993
12	11	hbo	before	10.43340234	4.189064506	2.490628235	34	0.01779771	0.0841635	0.47774266
12	12	hbo	before	1.52722492	4.110454487	0.371546486	34	0.712536	0.59621561	0.53366928
13	2	hbo	before	-4.222743424	2.66936635	-1.58192727	34	0.12292533	0.26168646	0.32671881
13	13	hbo	before	1.101145152	2.260236894	0.487181302	34	0.62925621	0.5576813	0.48691618
13	14	hbo	before	1.433892577	2.496806991	0.574290517	34	0.56955159	0.53361167	0.45967367
14	13	hbo	before	8.586986709	3.745379144	2.292688238	34	0.02817756	0.11199786	0.40048411
14	15	hbo	before	4.732125877	4.40016431	1.075442993	34	0.2897491	0.39180561	0.36901734
15	13	hbo	before	6.944962478	4.289897738	1.618910963	34	0.11470689	0.25562006	0.3245395
15	14	hbo	before	4.281802781	3.462543375	1.236606251	34	0.22470757	0.33878076	0.35242754
15	15	hbo	before	10.26741672	3.789461133	2.709466163	34	0.01047932	0.06545368	0.56406386
**15**	**16**	**hbo**	**before**	**13.25698986**	**4.236252923**	**3.129414155**	**34**	**0.00358463**	**0.03134536**	**0.71785288**
**16**	**1**	**hbo**	**before**	**9.629089108**	**3.220926429**	**2.989540221**	**34**	**0.00516306**	**0.03762315**	**0.66950273**
16	14	hbo	before	9.9213477	3.938026628	2.519370395	34	0.01662259	0.08385822	0.48910921
16	16	hbo	before	8.924803869	4.570367308	1.952754181	34	0.0591206	0.18463322	0.30830231
1	1	hbr	before	1.344880772	1.335935919	1.00669557	34	0.32119221	0.41167006	0.37736125
1	2	hbr	before	0.542114206	1.629527958	0.332681746	34	0.74141714	0.6045521	0.55313665
1	14	hbr	before	0.201608351	1.65198388	0.122040144	34	0.90358571	0.64926293	0.72341174
2	1	hbr	before	-0.603653651	1.230128015	-0.490724253	34	0.62677389	0.5576813	0.48569442
2	3	hbr	before	1.269607108	1.421518812	0.893134229	34	0.37806366	0.42492935	0.39326215
3	1	hbr	before	1.495419497	2.277425169	0.656627281	34	0.51584064	0.49928795	0.43837274
3	2	hbr	before	2.976681445	2.040328426	1.458922695	34	0.15376383	0.29229769	0.33466641
3	3	hbr	before	3.429531355	1.618660795	2.118746167	34	0.04149761	0.15119621	0.3357082
3	4	hbr	before	-1.117856988	1.428906251	-0.782316536	34	0.43944331	0.45300149	0.4119804
4	2	hbr	before	1.710107467	1.371140963	1.24721492	34	0.22084181	0.33682408	0.35145959
4	4	hbr	before	-1.291602134	1.950045413	-0.662344644	34	0.51221538	0.49928795	0.43702573
4	5	hbr	before	2.714993791	1.825463187	1.487290354	34	0.14615424	0.281918	0.33273114
5	3	hbr	before	1.294495569	1.3011961	0.994850483	34	0.3268369	0.41167006	0.37888917
5	4	hbr	before	-0.417861268	1.395311551	-0.299475245	34	0.7664006	0.60512218	0.57175082
5	6	hbr	before	0.065755533	1.366275254	0.048127588	34	0.96189616	0.66404097	0.84406895
6	4	hbr	before	0.606828524	1.639887511	0.370042774	34	0.71364579	0.59621561	0.53438109
6	5	hbr	before	1.249576381	1.479337852	0.844686276	34	0.40419078	0.4381224	0.40100368
6	6	hbr	before	0.568492866	1.358116135	0.418589288	34	0.67814877	0.58519644	0.51287691

6	7	hbr	before	0.136477194	2.472312437	0.055202244	34	0.95630046	0.663671	0.8292318
7	5	hbr	before	1.697137839	2.073705637	0.818408268	34	0.41882333	0.44662809	0.4054809
7	7	hbr	before	-0.156252149	1.384235783	-0.112879721	34	0.91078978	0.64926293	0.7352656
7	10	hbr	before	1.431594659	1.821021915	0.786149056	34	0.4372252	0.45300149	0.41127
8	6	hbr	before	0.310816648	1.392305604	0.223238812	34	0.82468549	0.62307219	0.62351891
8	7	hbr	before	1.67744152	1.296944666	1.293379404	34	0.20459858	0.33682408	0.34740224
9	7	hbr	before	3.147570629	2.785734963	1.129888762	34	0.26643148	0.36630939	0.36298575
**9**	**9**	**hbr**	**before**	**-8.643827645**	**2.105690936**	**-4.104984022**	**34**	**0.00023943**	**0.0083465**	**0.93547672**
9	10	hbr	before	-2.055687585	2.307385556	-0.890916379	34	0.37923548	0.42492935	0.39360286
10	9	hbr	before	-2.478373126	2.106028966	-1.176799164	34	0.24744888	0.3498848	0.3581521
10	10	hbr	before	-0.770844245	1.781033537	-0.43280726	34	0.66788798	0.5801589	0.5071068
10	11	hbr	before	0.88734046	1.584450845	0.560030286	34	0.57913025	0.53873777	0.46376588
10	12	hbr	before	-0.633558325	2.628469447	-0.241036975	34	0.81097413	0.62205842	0.61009534
11	5	hbr	before	0.909151245	4.614406735	0.197024514	34	0.84498144	0.62618581	0.64512864
11	10	hbr	before	0.487608476	1.526010713	0.319531489	34	0.75127866	0.6045521	0.56027051
11	12	hbr	before	0.827872885	1.689461271	0.490021819	34	0.62726569	0.5576813	0.4859358
12	11	hbr	before	-1.691581977	1.601914222	-1.055975379	34	0.29842539	0.39538561	0.37129335
12	12	hbr	before	2.586453486	2.059419553	1.255913824	34	0.2177093	0.33682408	0.35067604
13	2	hbr	before	-0.488621991	1.936937932	-0.252265177	34	0.8023549	0.61906731	0.60208641
13	13	hbr	before	0.143419689	1.372636744	0.10448481	34	0.91739857	0.65043906	0.74671642
13	14	hbr	before	1.911163198	1.589732847	1.202191426	34	0.23759562	0.34246589	0.35566446
14	13	hbr	before	1.426256913	2.141919401	0.665877956	34	0.50998193	0.49928795	0.43620107
14	15	hbr	before	2.740056985	1.977898437	1.385337555	34	0.17497127	0.31013823	0.3400055
15	13	hbr	before	3.247880049	2.007786347	1.617642263	34	0.11498115	0.25562006	0.32461278
15	14	hbr	before	-3.055836477	2.011399411	-1.51925891	34	0.13794274	0.27414176	0.33062627
15	15	hbr	before	-1.92506193	2.017614727	-0.954127616	34	0.3467538	0.415002	0.38436336
15	16	hbr	before	-2.703747043	2.397859237	-1.12756704	34	0.26739747	0.36630939	0.3632334
16	1	hbr	before	1.495075986	1.934624122	0.772799207	34	0.44498065	0.45300149	0.41376617
16	14	hbr	before	-1.173891948	1.734387062	-0.676833893	34	0.50309055	0.49928795	0.43368107
16	16	hbr	before	0.132355369	2.312099848	0.05724466	34	0.95468541	0.663671	0.82512952
1	1	hbo	after	4.840607456	2.838978856	1.70505231	34	0.09730708	0.24154138	0.31979437
1	2	hbo	after	9.372408727	3.563746453	2.629931408	34	0.01273582	0.06955145	0.53283315
1	14	hbo	after	5.06449392	2.70667283	1.871114182	34	0.06995553	0.2039059	0.31177856
**2**	**1**	**hbo**	**after**	**10.72424535**	**2.676948545**	**4.006145494**	**34**	**0.00031817**	**0.0083465**	**0.92287923**

2	3	hbo	after	-4.104199097	3.882681466	-1.057052744	34	0.29794054	0.39538561	0.37116566
3	1	hbo	after	0.709316295	5.993073486	0.118356015	34	0.90648207	0.64926293	0.72810278
3	2	hbo	after	-1.062296499	3.181162194	-0.33393346	34	0.74048075	0.6045521	0.55247292
3	3	hbo	after	-0.611057746	1.87646276	-0.325643418	34	0.74668987	0.6045521	0.55691782
3	4	hbo	after	-8.143383074	4.064342053	-2.003616568	34	0.05313221	0.18225447	0.3062709
4	2	hbo	after	7.821184456	2.992668285	2.613448505	34	0.01325641	0.06955145	0.52632746
4	4	hbo	after	-3.912049068	4.00551424	-0.976665874	34	0.3356329	0.41167006	0.38129033
4	5	hbo	after	0.535817424	3.227543429	0.166014009	34	0.86912921	0.63333301	0.67407651
5	3	hbo	after	-4.96012279	3.455217771	-1.435545635	34	0.16026833	0.3000041	0.3363112
5	4	hbo	after	4.874168859	2.861262207	1.703503037	34	0.09759939	0.24154138	0.31987587
5	6	hbo	after	4.446896811	2.860286829	1.554703104	34	0.12927705	0.26494866	0.32838196
6	4	hbo	after	2.291150757	3.698137291	0.619541833	34	0.53968935	0.51670607	0.44750678
6	5	hbo	after	-1.005894711	3.474357911	-0.2895196	34	0.77394201	0.60512218	0.57773977
6	6	hbo	after	-2.543529499	3.032777786	-0.838679811	34	0.40750694	0.4381224	0.40200901
6	7	hbo	after	-1.541038582	5.349827438	-0.288053886	34	0.7750542	0.60512218	0.57863869
7	5	hbo	after	-3.407904993	3.304815004	-1.031193876	34	0.30972986	0.40223697	0.37428805
7	7	hbo	after	4.161401609	2.362476667	1.761457231	34	0.08715237	0.22862804	0.31691622
7	10	hbo	after	4.552260195	3.765494599	1.208940838	34	0.23502591	0.34246589	0.35501768
8	6	hbo	after	-7.305723469	4.11208547	-1.776646795	34	0.08457553	0.22639609	0.31616974
8	7	hbo	after	-4.683090985	2.867290575	-1.633280919	34	0.11163787	0.25562006	0.3237166
9	7	hbo	after	-1.089247737	4.216584983	-0.258324626	34	0.79771376	0.61906731	0.59790146
9	9	hbo	after	1.373488149	3.354576677	0.409437101	34	0.68478699	0.58706252	0.51671057
9	10	hbo	after	-1.308093469	3.42523763	-0.381898604	34	0.70491316	0.59621561	0.52885259
10	9	hbo	after	-3.587355277	5.262277089	-0.681711589	34	0.50003904	0.49928795	0.43257684
10	10	hbo	after	5.523549218	5.349352224	1.032564129	34	0.30909718	0.40223697	0.37411953
10	11	hbo	after	5.92486989	4.493629032	1.318504453	34	0.19614808	0.33533744	0.34529457
10	12	hbo	after	-3.310281136	3.739670757	-0.885179833	34	0.38227724	0.42492935	0.39449001
11	5	hbo	after	3.276659362	3.511020278	0.933249911	34	0.35727158	0.415002	0.38731038
11	10	hbo	after	2.944680636	2.939653856	1.00170999	34	0.32355989	0.41167006	0.37800094
11	12	hbo	after	-12.19083318	5.182121143	-2.352479389	34	0.02457678	0.1039881	0.42354277
12	11	hbo	after	-11.91989397	4.529048942	-2.631875725	34	0.01267567	0.06955145	0.53359999
12	12	hbo	after	-8.725647571	4.401884409	-1.982252772	34	0.05557992	0.18225447	0.30711222
13	2	hbo	after	2.450375824	2.630933407	0.931371283	34	0.35822819	0.415002	0.38758046
13	13	hbo	after	-0.502928961	2.552914588	-0.197001875	34	0.84499902	0.62618581	0.64514835

13	14	hbo	after	3.323980615	2.656809572	1.251117374	34	0.21943237	0.33682408	0.35110696
14	13	hbo	after	-2.690563711	4.546648951	-0.591768518	34	0.55792029	0.52647486	0.45483181
14	15	hbo	after	-1.556598531	5.223090288	-0.29802252	34	0.76749962	0.60512218	0.57261226
15	13	hbo	after	-9.081514295	5.529299548	-1.642434854	34	0.10971836	0.25562006	0.32319916
15	14	hbo	after	1.00054106	5.83723319	0.171406731	34	0.86492006	0.63333301	0.6687428
15	15	hbo	after	-2.362911858	4.699687701	-0.502780612	34	0.61835993	0.5576813	0.48161545
15	16	hbo	after	3.099037157	4.740624429	0.653719189	34	0.51768992	0.49928795	0.43906394
**16**	**1**	**hbo**	**after**	**12.90160216**	**4.251283149**	**3.03475485**	**34**	**0.00459222**	**0.03762315**	**0.68551514**
**16**	**14**	**hbo**	**after**	**17.90290574**	**5.246549584**	**3.412319936**	**34**	**0.00167923**	**0.02002341**	**0.80337829**
16	16	hbo	after	9.447803388	4.823528625	1.958691266	34	0.05839265	0.18463322	0.30806008
1	1	hbr	after	1.446537548	1.473552984	0.981666464	34	0.33319835	0.41167006	0.38062321
1	2	hbr	after	-1.230521826	1.842957233	-0.667688758	34	0.50883937	0.49928795	0.43578071
1	14	hbr	after	2.439261139	1.802875455	1.352983719	34	0.18498964	0.32352395	0.34251036
2	1	hbr	after	2.114784608	1.286880986	1.643341251	34	0.10952978	0.25562006	0.3231482
2	3	hbr	after	3.129369143	1.513526708	2.067600873	34	0.04635568	0.16433177	0.31749714
3	1	hbr	after	3.88608686	2.952139072	1.316363073	34	0.19685774	0.33533744	0.34547156
3	2	hbr	after	7.015264094	2.486536386	2.821299594	34	0.00792942	0.05200339	0.60723424
3	3	hbr	after	2.720909385	1.940087544	1.402467323	34	0.16984098	0.30927101	0.33871948
3	4	hbr	after	3.026261216	1.573562365	1.923191151	34	0.06286356	0.18960654	0.30952929
4	2	hbr	after	1.914763637	1.485019326	1.289386342	34	0.20596671	0.33682408	0.34774357
4	4	hbr	after	0.07457994	2.108710244	0.035367562	34	0.97199348	0.6672201	0.87374628
4	5	hbr	after	1.015701376	2.038603176	0.498233981	34	0.6215269	0.5576813	0.48313962
5	3	hbr	after	3.73689019	1.410097408	2.650093652	34	0.01212472	0.06955145	0.54077839
5	4	hbr	after	2.998935027	1.563963393	1.91752252	34	0.06360419	0.18960654	0.3097686
5	6	hbr	after	2.485945994	1.457042057	1.706159394	34	0.09709864	0.24154138	0.31973621
6	4	hbr	after	0.970595035	1.831134966	0.530051063	34	0.59952131	0.54990652	0.47281442
6	5	hbr	after	1.282405498	1.651656935	0.776435755	34	0.44285995	0.45300149	0.41308016
6	6	hbr	after	-0.447305104	1.449262382	-0.308643286	34	0.75947616	0.60512218	0.56640926
6	7	hbr	after	5.789112474	2.913992911	1.986659766	34	0.05506711	0.18225447	0.30693728
7	5	hbr	after	1.222156677	2.036320246	0.600179014	34	0.55236657	0.52501122	0.45256713
7	7	hbr	after	1.269027327	1.449637196	0.875410296	34	0.38749333	0.42710785	0.3960207
**7**	**10**	**hbr**	**after**	**6.191957765**	**2.060008894**	**3.005791762**	**34**	**0.00495058**	**0.03762315**	**0.67529767**
8	6	hbr	after	1.032403146	1.337530262	0.771872739	34	0.4455219	0.45300149	0.41394168
8	7	hbr	after	-0.11411921	1.412770363	-0.080776899	34	0.936093	0.65769114	0.7826445

9	7	hbr	after	4.925759275	2.243526919	2.195542756	34	0.03504784	0.13520809	0.36381642
9	9	hbr	after	3.706557651	2.189750773	1.692684709	34	0.09966109	0.24207624	0.32044877
9	10	hbr	after	5.603122152	2.299727893	2.436428314	34	0.02022263	0.09146606	0.45636766
10	9	hbr	after	-0.44133654	2.19192345	-0.201346694	34	0.84162726	0.62618581	0.64140068
10	10	hbr	after	-0.055859428	1.896518276	-0.029453673	34	0.97667498	0.6672201	0.88909384
10	11	hbr	after	0.599905428	1.829228314	0.327955468	34	0.74495642	0.6045521	0.55566645
10	12	hbr	after	-0.247068404	3.135479156	-0.078797655	34	0.93765551	0.65769114	0.78592209
11	5	hbr	after	9.882982391	4.237519827	2.332256318	34	0.02574525	0.10552792	0.41570853
11	10	hbr	after	1.397504293	1.43146916	0.976272722	34	0.33582481	0.41167006	0.381343
**11**	**12**	**hbr**	**after**	**6.12436357**	**1.906878907**	**3.211721284**	**34**	**0.00288268**	**0.02908529**	**0.74454008**
12	11	hbr	after	2.732505301	1.729650684	1.579801822	34	0.12341189	0.26168646	0.32684682
12	12	hbr	after	6.177879543	2.601133734	2.375071863	34	0.02332833	0.10199589	0.43233234
13	2	hbr	after	-0.606140067	1.783088798	-0.339938239	34	0.73599423	0.6045521	0.5493246
13	13	hbr	after	-0.726344653	1.515577424	-0.479252753	34	0.63482709	0.55884255	0.48968942
13	14	hbr	after	0.755424082	1.743773127	0.433212366	34	0.66759656	0.5801589	0.50694559
14	13	hbr	after	3.227489494	2.662324038	1.212282746	34	0.23376118	0.34246589	0.35469962
14	15	hbr	after	0.413444782	2.422879264	0.170641925	34	0.86551677	0.63333301	0.66949101
15	13	hbr	after	-2.212065998	2.375745221	-0.93110405	34	0.3583644	0.415002	0.38761895
15	14	hbr	after	-1.584743327	2.863463106	-0.553435916	34	0.58358629	0.5390599	0.4657032
15	15	hbr	after	0.496449483	2.143824543	0.231571882	34	0.81825861	0.62307219	0.61711784
15	16	hbr	after	2.23358354	2.401811342	0.929957945	34	0.35894897	0.415002	0.3877842
16	1	hbr	after	4.281834651	2.39985992	1.784201909	34	0.08331799	0.22639609	0.3158028
16	14	hbr	after	3.516203723	1.93267271	1.819347738	34	0.07767415	0.21676988	0.31413271
16	16	hbr	after	4.068497772	2.660472181	1.529238983	34	0.13545666	0.27334259	0.32998504

Supplemental Table 6. Numerical results of the general linear model (GLM)-based statistical approach to detect differences in neurovascular coupling (NVC) assessed by fNIRS. Rows highlighted with bold font indicate significantly different NVC when comparing NVC prior to (pre-SD) and after (post-SD) 24 hours of sleep deprivation.

source	detector	Hb type	cond	beta	se	tstat	dfe	p	q	power
1	1	hbo	-[post-SD]+[pre-SD]	-0.98419152	3.3682164	-0.2922	34	0.77191	0.871804	0.5761077
1	2	hbo	-[post-SD]+[pre-SD]	1.349352422	4.0500282	0.3331711	34	0.741051	0.846915	0.5528769
1	14	hbo	-[post-SD]+[pre-SD]	-5.39306189	3.1412891	-1.716831	34	0.095109	0.276679	0.3191791
**2**	**1**	**hbo**	-[post-SD]+[pre-SD]	**-10.2147855**	**3.1396387**	**-3.253491**	**34**	**0.002579**	**0.024755**	**0.7575367**
2	3	hbo	-[post-SD]+[pre-SD]	8.212220027	4.6002465	1.7851696	34	0.083158	0.275282	0.315756
3	1	hbo	-[post-SD]+[pre-SD]	7.874552144	7.2429295	1.0872054	34	0.284594	0.534359	0.3676735
3	2	hbo	-[post-SD]+[pre-SD]	9.24256151	3.6223484	2.5515385	34	0.015392	0.092352	0.5018412
**3**	**3**	**hbo**	-[post-SD]+[pre-SD]	**6.866485135**	**1.8736694**	**3.664726**	**34**	**0.000837**	**0.011475**	**0.864021**
**3**	**4**	**hbo**	-[post-SD]+[pre-SD]	**25.49596935**	**4.7723063**	**5.3424838**	**34**	**6.17E-06**	**0.000592**	**0.9956567**
4	2	hbo	-[post-SD]+[pre-SD]	-1.92251487	3.2239539	-0.596322	34	0.55491	0.764427	0.4536006
**4**	**4**	**hbo**	-[post-SD]+[pre-SD]	**21.15817127**	**5.1979009**	**4.0705222**	**34**	**0.000264**	**0.008462**	**0.9312887**
**4**	**5**	**hbo**	-[post-SD]+[pre-SD]	**11.85490078**	**3.5598168**	**3.3301997**	**34**	**0.002098**	**0.022381**	**0.7803985**
**5**	**3**	**hbo**	-[post-SD]+[pre-SD]	**14.09741499**	**3.6347816**	**3.8784765**	**34**	**0.000458**	**0.008795**	**0.9037983**
5	4	hbo	-[post-SD]+[pre-SD]	3.958176317	3.1910045	1.240417	34	0.223313	0.459497	0.3520783
5	6	hbo	-[post-SD]+[pre-SD]	-1.62845142	3.3829256	-0.481374	34	0.633335	0.800002	0.4889422
6	4	hbo	-[post-SD]+[pre-SD]	5.473413549	4.2376528	1.2916144	34	0.205202	0.458126	0.3475529
6	5	hbo	-[post-SD]+[pre-SD]	1.763997462	4.1250437	0.4276312	34	0.671616	0.819658	0.5091819
6	6	hbo	-[post-SD]+[pre-SD]	-0.41183963	3.5930637	-0.114621	34	0.90942	0.959959	0.7329631
6	7	hbo	-[post-SD]+[pre-SD]	-11.1381947	6.0981974	-1.826473	34	0.07657	0.262527	0.3138013
7	5	hbo	-[post-SD]+[pre-SD]	6.533225901	4.458697	1.465277	34	0.152033	0.364878	0.3342272
7	7	hbo	-[post-SD]+[pre-SD]	-1.13135731	2.712585	-0.417077	34	0.679244	0.819658	0.5135037
7	10	hbo	-[post-SD]+[pre-SD]	-0.97166795	4.5182429	-0.215054	34	0.83101	0.906556	0.6300166
8	6	hbo	-[post-SD]+[pre-SD]	3.031939449	4.7230174	0.6419497	34	0.525211	0.763043	0.4419039
8	7	hbo	-[post-SD]+[pre-SD]	2.436638356	3.1544164	0.772453	34	0.445183	0.712293	0.4138317
9	7	hbo	-[post-SD]+[pre-SD]	7.224635593	6.0210071	1.1999048	34	0.238471	0.476942	0.3558849
9	9	hbo	-[post-SD]+[pre-SD]	1.782902316	4.456244	0.4000908	34	0.691592	0.819665	0.5207266
9	10	hbo	-[post-SD]+[pre-SD]	4.112613478	4.3026433	0.9558342	34	0.345903	0.603758	0.3841268
10	9	hbo	-[post-SD]+[pre-SD]	10.24336974	6.3618268	1.6101303	34	0.116616	0.310976	0.3250488
10	10	hbo	-[post-SD]+[pre-SD]	12.16733049	6.8886077	1.7662975	34	0.086324	0.276237	0.3166771
10	11	hbo	-[post-SD]+[pre-SD]	3.069141481	5.6304648	0.5450956	34	0.589246	0.764427	0.4681956
10	12	hbo	-[post-SD]+[pre-SD]	7.630874183	4.4306338	1.7222986	34	0.094102	0.276679	0.3188961
11	5	hbo	-[post-SD]+[pre-SD]	2.367145853	5.1675273	0.458081	34	0.649809	0.810151	0.4973712
11	10	hbo	-[post-SD]+[pre-SD]	-0.31754003	3.7776214	-0.084058	34	0.933503	0.959959	0.7773143

11	12 hbo	-[post-SD]+[pre-SD]	16.11931866	6.1919843	2.6032558	34	0.013588	0.086964	0.5223006
**12**	**11 hbo**	-[post-SD]+[pre-SD]	**22.46599674**	**5.6846062**	**3.9520762**	**34**	**0.000371**	**0.008795**	**0.9152003**
12	12 hbo	-[post-SD]+[pre-SD]	10.4620664	5.5392676	1.8887093	34	0.067486	0.259147	0.3110057
13	2 hbo	-[post-SD]+[pre-SD]	-6.63383448	3.0465836	-2.177467	34	0.03648	0.152264	0.3571239
13	13 hbo	-[post-SD]+[pre-SD]	1.694084115	2.9993002	0.5648265	34	0.5759	0.764427	0.4623749
13	14 hbo	-[post-SD]+[pre-SD]	-1.81882547	3.2994177	-0.551257	34	0.585063	0.764427	0.4663499
14	13 hbo	-[post-SD]+[pre-SD]	11.58251767	5.4223867	2.1360553	34	0.039959	0.159834	0.341967
14	15 hbo	-[post-SD]+[pre-SD]	6.58545602	6.1917262	1.0635897	34	0.295011	0.534359	0.3703953
15	13 hbo	-[post-SD]+[pre-SD]	16.06574375	6.5170374	2.4651913	34	0.018901	0.100803	0.4676987
15	14 hbo	-[post-SD]+[pre-SD]	3.682898686	6.5317554	0.5638452	34	0.57656	0.764427	0.4626583
15	15 hbo	-[post-SD]+[pre-SD]	12.69554567	5.4839774	2.3150252	34	0.02678	0.128545	0.4090608
15	16 hbo	-[post-SD]+[pre-SD]	10.35150746	5.9623074	1.736158	34	0.091591	0.276679	0.318186
16	1 hbo	-[post-SD]+[pre-SD]	-3.21138489	5.092861	-0.630566	34	0.53254	0.763043	0.4447174
16	14 hbo	-[post-SD]+[pre-SD]	-7.84237175	6.3454065	-1.235913	34	0.224962	0.459497	0.3524912
16	16 hbo	-[post-SD]+[pre-SD]	-0.21301469	6.3905754	-0.033333	34	0.973604	0.983853	0.8788964
1	1 hbr	-[post-SD]+[pre-SD]	-0.12879398	1.181351	-0.109023	34	0.913825	0.959959	0.7404535
1	2 hbr	-[post-SD]+[pre-SD]	1.767023431	1.6048752	1.1010348	34	0.278616	0.534359	0.3661229
1	14 hbr	-[post-SD]+[pre-SD]	-2.2115355	1.5257119	-1.449511	34	0.156357	0.366104	0.3353231
**2**	**1 hbr**	-[post-SD]+[pre-SD]	**-2.85816559**	**0.753985**	**-3.790746**	**34**	**0.000587**	**0.009397**	**0.8887068**
2	3 hbr	-[post-SD]+[pre-SD]	-1.83543449	1.1910762	-1.540988	34	0.132576	0.326342	0.3292395
3	1 hbr	-[post-SD]+[pre-SD]	-2.25598244	3.3022951	-0.683156	34	0.499137	0.763043	0.4322519
3	2 hbr	-[post-SD]+[pre-SD]	-3.99460645	2.3736511	-1.682895	34	0.101558	0.285931	0.3209729
3	3 hbr	-[post-SD]+[pre-SD]	0.641376212	1.8497525	0.3467362	34	0.730926	0.845409	0.5458302
**3**	**4 hbr**	-[post-SD]+[pre-SD]	**-4.17498594**	**1.1769262**	**-3.547364**	**34**	**0.001159**	**0.013911**	**0.8377072**
4	2 hbr	-[post-SD]+[pre-SD]	-0.20696617	0.9597646	-0.215643	34	0.830555	0.906556	0.6295424
4	4 hbr	-[post-SD]+[pre-SD]	-1.38822859	1.673614	-0.82948	34	0.412619	0.682956	0.4035694
4	5 hbr	-[post-SD]+[pre-SD]	1.7052071	2.0057764	0.8501481	34	0.40119	0.675688	0.4000985
5	3 hbr	-[post-SD]+[pre-SD]	-2.3898369	1.0733384	-2.226546	34	0.032708	0.142726	0.375396
5	4 hbr	-[post-SD]+[pre-SD]	-3.40675731	1.2167665	-2.799845	34	0.008369	0.061798	0.5990405
5	6 hbr	-[post-SD]+[pre-SD]	-2.30977389	1.263374	-1.828258	34	0.076296	0.262527	0.3137187
6	4 hbr	-[post-SD]+[pre-SD]	-0.61126146	1.697159	-0.360167	34	0.72095	0.844039	0.539135
6	5 hbr	-[post-SD]+[pre-SD]	0.009725871	1.2983528	0.0074909	34	0.994067	0.994067	0.9611574
6	6 hbr	-[post-SD]+[pre-SD]	1.067407546	1.2387971	0.8616484	34	0.394917	0.675688	0.3982203
6	7 hbr	-[post-SD]+[pre-SD]	-5.67555456	3.4000006	-1.66928	34	0.104246	0.285931	0.3217112

7	5	hbr	-[post-SD]+[pre-SD]	0.454154925	1.9519734	0.2326645	34	0.817417	0.906556	0.616294
7	7	hbr	-[post-SD]+[pre-SD]	-1.40870564	1.113706	-1.264881	34	0.214515	0.459497	0.3498777
7	10	hbr	-[post-SD]+[pre-SD]	-4.72822653	1.9124377	-2.472356	34	0.018584	0.100803	0.4705258
8	6	hbr	-[post-SD]+[pre-SD]	-0.66050586	1.1162139	-0.591738	34	0.557941	0.764427	0.4548402
8	7	hbr	-[post-SD]+[pre-SD]	1.79119626	1.1525317	1.5541405	34	0.129411	0.326342	0.3284169
9	7	hbr	-[post-SD]+[pre-SD]	-1.7806228	2.9633706	-0.600878	34	0.551907	0.764427	0.4523809
**9**	**9**	**hbr**	-[post-SD]+[pre-SD]	**-12.3733655**	**2.4260599**	**-5.10019**	**34**	**1.27E-05**	**0.000611**	**0.9921807**
9	10	hbr	-[post-SD]+[pre-SD]	-7.63951045	2.7214252	-2.807173	34	0.008216	0.061798	0.6018444
10	9	hbr	-[post-SD]+[pre-SD]	-1.95009996	2.4798634	-0.786374	34	0.437095	0.711206	0.4112285
10	10	hbr	-[post-SD]+[pre-SD]	-0.79953298	1.94141	-0.411831	34	0.683048	0.819658	0.5156985
10	11	hbr	-[post-SD]+[pre-SD]	0.334017323	1.6963644	0.1969019	34	0.845077	0.911543	0.6452354
10	12	hbr	-[post-SD]+[pre-SD]	-0.2729938	3.4667983	-0.078745	34	0.937697	0.959959	0.7860096
11	5	hbr	-[post-SD]+[pre-SD]	-8.88304691	5.5941639	-1.587913	34	0.121563	0.315408	0.3263599
11	10	hbr	-[post-SD]+[pre-SD]	-1.0011661	1.4423126	-0.694139	34	0.492311	0.763043	0.4298113
11	12	hbr	-[post-SD]+[pre-SD]	-5.32324729	1.8114665	-2.93864	34	0.005886	0.051365	0.651081
12	11	hbr	-[post-SD]+[pre-SD]	-4.38680161	1.6182451	-2.710839	34	0.010444	0.071615	0.5645998
12	12	hbr	-[post-SD]+[pre-SD]	-3.57283746	2.6875561	-1.3294	34	0.192568	0.440154	0.3444015
13	2	hbr	-[post-SD]+[pre-SD]	0.109870815	1.4479718	0.0758791	34	0.93996	0.959959	0.7908445
13	13	hbr	-[post-SD]+[pre-SD]	0.881264877	1.3781274	0.6394654	34	0.526806	0.763043	0.4425122
13	14	hbr	-[post-SD]+[pre-SD]	1.186379815	1.761471	0.6735165	34	0.505172	0.763043	0.4344383
14	13	hbr	-[post-SD]+[pre-SD]	-1.77099009	2.7647467	-0.640561	34	0.526102	0.763043	0.4422434
14	15	hbr	-[post-SD]+[pre-SD]	2.364325704	2.291327	1.0318587	34	0.309423	0.550085	0.3742062
15	13	hbr	-[post-SD]+[pre-SD]	5.420972843	2.42082	2.2393127	34	0.031786	0.142726	0.3801995
15	14	hbr	-[post-SD]+[pre-SD]	-1.54736293	2.9249836	-0.529016	34	0.600231	0.768296	0.4731382
15	15	hbr	-[post-SD]+[pre-SD]	-2.34782911	2.1876511	-1.073219	34	0.290731	0.534359	0.369274
15	16	hbr	-[post-SD]+[pre-SD]	-4.92522778	2.6749142	-1.841266	34	0.074322	0.262527	0.313121
16	1	hbr	-[post-SD]+[pre-SD]	-2.73121511	2.5650429	-1.064783	34	0.294478	0.534359	0.3702554
16	14	hbr	-[post-SD]+[pre-SD]	-4.74887487	1.9970016	-2.378003	34	0.023171	0.117073	0.4334752
16	16	hbr	-[post-SD]+[pre-SD]	-3.83312025	3.0394025	-1.261143	34	0.215842	0.459497	0.3502094
